# Are Smart Cities ‘Sustainable?’ Revisiting the ‘Helix Model’ of the ASEAN Smart Cities Network

**DOI:** 10.12688/f1000research.174616.1

**Published:** 2025-12-31

**Authors:** Bama Andika Putra

**Affiliations:** 1 International Relations, Hasanuddin University Faculty of Social and Political Sciences, Makassar, South Sulawesi, Indonesia; 2 University of Bristol School of Sociology Politics and International Studies, Bristol, England, UK

**Keywords:** Smart Cities, ASEAN, Helix Model, Sustainability, Southeast Asia

## Abstract

Recent urban planning in Southeast Asia has incorporated the theme of transforming cities to ‘smart city’ status. However, the central question raised in this study is whether the ASEAN’s conception of smart cities is genuinely sustainable. Based on the backdrop of Southeast Asia’s disparity in economic performances and diversity in its political, social, economic, and environmental dimensions, this study updates Crumpton’s 2021 study on the ‘quintuple helix model’ and argues that new observations but old patterns are found in the ASEAN Smart Cities Network’s way of developing cities in Southeast Asia. Utilizing qualitative and quantitative methods and considering data among the ten ASEAN member states in the past four years, this study revisits and updates the current state of Southeast Asia and how this corresponds to the challenges of constructing smart cities in democratic, semi-authoritarian, and authoritarian settings.

## 1. Introduction

Are smart cities sustainable? Past studies have doubted that smart city approaches taken by local governments lead to urban sustainability. ^1– 5^ In recent years, we have seen the trend of cities adopting smart city grand strategy schemes as a means to accelerate growth. Nevertheless, how many compromises are made to achieve the present and future needs?

Among the regions that have actively adopted this smart city conception is the Southeast Asian Region. Under the Association of Southeast Asian Nations (ASEAN), particularly during Singapore’s ASEAN chairmanship in 2018, member states agreed with a list of 26 pilot projects under the ASEAN Smart Cities Network (ASCN). ^6^ The growth of cities in ASEAN is unique, as they will “continue to be driven by urban centers, with more people expected to urbanize by 2030 and ‘middleweight’ cities of between 200,000 and 2 million residents forecast to drive 40% of the region’s growth”. ^7^ The role of ASCN is thus to act as a normative guideline for ASEAN member states on smart city development, sharing best practices, and linking partnerships to accelerate growth. ^6, 8^ The expectation is that the ASCN will act as the facilitator to link city government officials and business entities to “catalyze bankable projects to solve urban problems by utilizing technology and innovation opportunities provided by the private sector”. ^9^

For ASEAN, the strategic outcomes of smart cities consist of a high quality of life, a competitive economy, and a sustainable environment. ^6^ To achieve them, several development focus areas have been determined: civic and social, health and well-being, safety and security, quality environment, built infrastructure, and industry and innovations. The determined development areas are enabled through ASEAN’s encouragement of digital infrastructure and applications and partnership and funding schemes under the ASCN. ^10^

Scholars have interpreted the ASCN in different ways. The most common discourse introduced is how the active participation of local governments in offering and seeking partnerships as a representation of ‘paradiplomacy’ as non-central government actors actively engage in international relations. ^7, 9, 11, 12^ The interlinkages between cities and business entities have also led some studies to conclude the presence of ‘transboundary learning’ in the ASCN. ^13, 14^ Nevertheless, the more recent discourse aims to connect sustainability with the smart city conceptions of the ASCN, with studies focusing on the impacts on tourism, human rights, and governance. ^12, 15, 16^

This article is interested in updating the data of Charles David Crumpton’s ‘quintuple helix model’ to assess the nexus between the ASCN and sustainability. The approach is grounded by the ‘helix models’ that symbolize the interactions among different sectors. ^17, 18^ The interactions considered include actors in the education, entrepreneurial, public, media, and ecological contexts, ^19– 23^ which collectively can boost regional innovation systems. The Crumpton-led 2021 study concluded that the diversity found in Southeast Asian states, some consisting of authoritarian systems, has severely impeded the “socio-ecologically sustainable urban planning and governance” of the ASCN’s smart cities. ^20^ In the ASCN’s development focus area of civic and social, for example, some countries, such as Vietnam, Laos, Cambodia, and Brunei Darussalam, still lack the basic features of democracy within their countries. ^24– 26^ Thus, the intention of its cities to transform into smart cities is seen as a potential policy to further impinge upon democratic voices. ^20^

Through quantitative and qualitative approaches, this study intends to provide an update on the ASCN under the ‘helix models.’ It considers several unique trends between 2021 and 2024 that contribute to the diversity of Southeast Asian states. These include the Myanmar military coup in 2021, Laos’ foreign debt surges, and Cambodia’s change of leadership after more than three decades, to name a few. ^27– 33^

The recent developments since 2021 reveal a more complex region in Southeast Asia, making it convoluted to introduce sustainability measures. In its current form, most Southeast Asian states are sensitive to the notion of imposed regulations and systems, as the region is founded on the norm of non-interference. ^34, 35^ Consequently, through ASEAN, Southeast Asian states agree that introducing new norms into the region will be undertaken at a slow pace, one that caters to the unique differences in the region. In addition, ASEAN’s consensus-based decision-making system only allows the organization to adopt mechanisms and regulations if no rejections are made by even one of its member states. Some unique trends further exacerbate such a unique context to Southeast Asia’s politics. In many parts of Southeast Asia, there is a visibly more apparent dependence upon China’s Belt and Road Initiative (BRI), which, unfortunately, has led to the increase of foreign debt for the smaller Southeast Asian states. ^36– 39^ Democracy is another prominent problem in the region. With Myanmar’s undemocratic rule and questions over Cambodia’s stability due to leadership changes, there are questions about whether Southeast Asia can put aside differences to undertake sustainability measures. In the past, pressing domestic challenges and a lack of democracy also contributed to the lack of the region’s decisive measures to provide human rights protection at an ASEAN-region level. ^40– 42^

Consequently, these changes within the political landscape of Southeast Asian states make the inquiry of sustainability in smart cities particularly interesting to re-explore. Inspired by Crumpton’s analysis in 2021, this study will provide the ‘helix model’s’ interpretation of the ASCN with the following differences. First is the inclusion of more recent data (between 2021 and 2024) that incorporates differences in the political, social, environmental, and economic landscape of Southeast Asian states. Second is the exclusion of one of the supposed helix models of interactions among educational stakeholders due to the inconclusive relationship between information and communication technology projects and having high-ranked universities. ^20^ Third is the inclusion of the Ease of Doing Business (EoDB) rankings to complement past data on the differences in the Southeast Asian economic landscape. The intention of this study is thus similar to past studies, which aim to reveal the profoundly diverse region of Southeast Asia, which poses challenges to imposing sustainability measures in the ASCN’s conception of smart cities.

## 2. The ASEAN, ASCN, Smart Cities, and Sustainability: A Literature Review

Against the backdrop of the unique norms and differences among states, adopting sustainability measures in Southeast Asia comes with challenges. The existing literature highlights those challenges well, focusing on the trajectory of sustainability of ASEAN member states in the past years. Nevertheless, not enough interpretations are made on ASEAN’s development pathway, especially with its recent emphasis on doing so through the means of constructing smart cities across the region. This section provides a brief literature review of how Southeast Asia engages with sustainability demands. First, it looks into the challenges of adopting sustainability in different regional sectors and how there has been a tendency within the literature to call for modified governance and policies to address the concerns. Second, this section covers how the ASCN has been one of ASEAN’s solutions to address issues related to urbanization in Southeast Asia’s larger cities, primarily looking into smart city initiatives as the basis for its solution. Last, it will revisit the recent studies on smart cities and sustainability in Southeast Asia and the novelty offered by this study.

### 2.1 Sustainability in Southeast Asia: Conflicting Norms?

Perhaps the primary discourse relevant to this study is studies exploring the sustainability element of Southeast Asian states’ practices across time. Within this discourse, studies have been particularly critical in questioning whether Southeast Asian states have ensured sustainable measures are in place concerning the vast development projects. Based on an evaluation of different sectors, scholars agree that Southeast Asia still encounters issues with converging sustainability measures with its development practices. Ruland, for example, argued that path dependencies and development practices are the reason Southeast Asia’s hydropower dams lack elements of environmental sustainability. ^43^ Also, in the energy sector, scholars have looked into how carbon emissions in Southeast Asian states have faced an increase in volume due to the region’s focus on economic development and the encountered challenges of inadequate infrastructure and limitation of technologies. ^44^ Linked to the field of tourism, other studies have pointed to Southeast Asia’s tourism policies as lacking consideration of potential environmental elements, which consequently leads to unsustainable practices. ^45^ As Putra rightfully concludes when assessing the politics of countering climate change in the region, Southeast Asia’s unique norms of non-interference and persistence in undertaking development practices to advance their respective nations have equally contributed to the lack of sustainability practices in the region. ^46^

Although the highlight of challenges has been the dominant discourse, there have been some studies looking into what the ideal solution could be. Studies have argued that policymakers should re-evaluate their good governance to ensure environmental sustainability. ^47^ Others have argued the nexus between corporate governance and corporate sustainability disclosures, arguing that it is the baseline for ensuring that development in the region is balanced with careful consideration of the environment. ^48^ However, other studies have also pointed out that the issue is multidimensional and needs simultaneous approaches. Dai, for example, in a recently published article, called for the following measures to be adopted: tax incentives, financial institution collaborations, ESG investing, and transparency. ^49^

Another discourse worth mentioning is the development gap between ASEAN member states. The challenges and potential solutions outlined in past studies are common problems all states face. Nevertheless, finding a balanced solution for ASEAN has been challenging for its member states due to the disparities in the capacity between Southeast Asian states. Past studies have highlighted The problem well. ^50– 52^ Most have looked into the newer states of ASEAN, oftentimes termed as CMLV (Cambodia, Myanmar, Laos, Vietnam), as these states still lack resources and supporting systems to strive for a balanced development approach. ^53^ Consequently, the problem of sustainability is enhanced when discussing such states. Nevertheless, no direct connections are made to how, for example, the lack of democratic practices in those states (or any Southeast Asian state) would impact its sustainability practices. As part of this solution, ASEAN established the ASCN, focusing on city actors as the basis of its solution. How has this been interpreted in existing studies?

### 2.2 Is the ASCN the Solution to the Slow Performing Sustainability Measures in the Region?

With the challenges associated with development and sustainability prominent in many parts of Southeast Asia, the ASCN has been one of ASEAN’s solutions. One of the reasons this has surfaced is due to urbanization-related challenges, with cities being the primary stakeholders that can make direct changes. Arfanuzzaman and Dahiya, for example, argue that urbanization in the region is uncontrollable due to its haphazard nature. ^54^ A study in 2020 and 2021 found that the urbanization challenges faced in Asia mimic that of Western cities, with the larger cities of Southeast Asia, such as Kuala Lumpur, Jakarta, and Manila, encountering the challenge of population densities. ^55, 56^ These more recent studies are consistent with the earlier findings of Giok Ling Ooi in 2008 and 2009. Her studies predicted that Southeast Asian cities would face in-migration and urban sprawl, leading to environmental degradation over the long run. ^57^ In her 2009 study, she argued that such states fail to “[…] identify and implement an urban development policy framework that will link the effort at more sustainable development in a variety of sectors”. ^58^

Nevertheless, the city-oriented solution is not only due to issues of urbanization. Studies have looked into the issue of decentralization as one of the primary factors influencing urban resilience. Marks and Pulliat looked into how fragmented governance structures and “misaligned incentive structures” are causing uneven commitment among cities to tackle climate change. ^59^ Amid the rise of such challenges, scholars have looked into different conceptions to alleviate the impact of the lack of sustainability measures adopted within Southeast Asian states. These include an inquiry into the potential of smart cities being adopted in the region and the potential development of smart cities as the foundation for constructing sustainable cities. ^15, 60^

Established in 2018, the ASCN has quickly attracted the attention of scholarly inquiries, questioning whether the ASCN would contribute to developing more sustainable cities. Most studies have concluded in favor of the initiative, arguing that the ASCN would catalyze partnerships and help the region with its digitization efforts. ^9, 61^ Others have looked at the advantages of the ASCN due to its potential to establish transboundary learning ^14^ and “[…] fostering autonomy-enhancing initiatives between developing countries that have the capacity to learn from and scale-up locally-informed, adaptive problem solving”. ^16^ Nevertheless, some studies have been critical in addressing the ASCN. Two concerns have been prominent: First is the impact of ‘technocratic regionalism,’ defined as the technologically-driven regional integration resulting from the ASCN, ^62^ and second, how achieving the ‘smart city’ status would not be able to be balanced with human rights concerns that have already become a significant challenge for many Southeast Asian states. ^12^ Based on that backdrop, it is clear that the smart city destination the ASCN aims to achieve for its participating cities can generate a mixture of positive and negative outcomes. The following subsection will examine how past studies have interpreted such a dilemma and elaborate on the novelty offered in this updated study.

### 2.3 Revisiting Discussions on Smart Cities and Sustainability in Southeast Asia

Studies in the past have captured the doubt that smart cities are indeed sustainable means of developing cities. As past studies have shown, there is unclear evidence that shows that the smart city approach indeed leads to urban sustainability. ^1, 3, 5, 63, 64^ In building up such an assumption, Charles David Crumpton, Supawatanarkorn Wongthanavasau, Peerasit Kamnuansilpa, John Draper, and Eva Bialobrzeski specifically analyzed this in the context of the ASCN. Adopting the ‘helix models,’ the study looks into specific city plans in Southeast Asia, the ASCN, and experiences with the smart city initiative. ^20^ The study highlights the disparities in social, economic, and political rights among Southeast Asian states and argues that the presence of states with authoritarian settings generates a more significant challenge for the ASCN. It is then proposed that the smart city conceptualization to be within a ‘system of systems’ framework. ^20^ In this, “the complex socio-ecological interaction between society and nature and the multi-layered character of governance are acknowledged”. ^20^

Consequently, the disparity in economic capacity, social and human rights, and political systems between the Southeast Asian states are perceived as barriers to achieving the ideal smart city status for ASCN participating cities. With the advancement of technologies proposed as the basis of a smart city, ASEAN is bridging the potential control abuses and heightened cyber surveillance that could be the case in many of the authoritarian nations in the region. As Crumpton’s-led study mentioned, “[…] authoritarian regimes and regimes with authoritarian tendencies seek new and better means for surveilling and controlling their populations”. ^20^ And that such state systems would impact “[…] participatory and therefore socio-ecologically sustainable urban planning and governance”. ^20^

This study builds up the previous concerns on the nexus between smart cities and sustainability in Southeast Asia. Within 2021 and 2024, many developments have taken place that contribute to the issue of capacity disparity, political tensions, and social and human rights being impeded. Unfortunately, as a means to balance development and sustainability, ASEAN has focused on the ASCN as one of the primary means of achieving a balanced development for the region’s larger cities. The novelty of this study, thus, offers to reveal the profound problems associated with the ASCN, which distances itself from actual sustainability measures as the primary aim and further impacts participatory and socio-ecological sustainable urban planning and governance. In doing so, it follows the framework introduced in 2019 and points out how the region’s socio-political developments have contributed to the disparities.

## 3. The ‘Helix Model’ of the ASCN: New Observations, Old Patterns

### 3.1 Recent Developments of the ASCN

The original plan of the ASCN is threefold: First, it will facilitate cooperation on smart city development, catalyze bankable projects, and secure funding from ASEAN’s external partners. ^65^ Established during Singapore’s ASEAN chairmanship in 2018, ASEAN envisioned that despite differences in political systems and economic landscape among the member states, the vision to develop smart cities within Southeast Asia would be perceived as a priority for local governments. As of September 2024, the total number of ASCN’s smart city projects accumulates to 108. ^6^ Divided into the development areas of the ASCN, 27% of projects are civic and social, 18% on quality environment, 6% on health and well-being, 26% on built infrastructure, 12% on safety and security, and 11% on industry and innovation. ^6^ At the core of facilitating smart city development, the ASCN Smart Cities Framework continues to articulate the essential features that ASEAN cities aim to achieve.

Cities incorporated into the ASCN have expanded. The pilot ASCN cities initially incorporated 26 cities from the ten ASEAN members. Since June 2024, the list has grown to include five new cities, including Chiang Mai (Thailand), Khon Kaen (Thailand), Rayong (Thailand), Sumedang (Indonesia) and Sihanoukville (Cambodia). ^10^ Already in this list of ASCN are notable Southeast Asian capital cities such as Jakarta, Bangkok, Manila, Kuala Lumpur, and Singapore. These cities have seen a rapid rise in urbanization, with common issues faced in their public services. ^66– 69^ The challenges of urbanization, employment opportunities, environmental degradation, people’s welfare, and the lack of adequate transportation facilities are common problems among the pilot ASCN cities. ^9, 12, 16, 70^

In recent years, Southeast Asian cities have actively engaged in paradiplomacy to secure funding from ASEAN’s external partners. Since 2018, members of the East Asia Summit (EAS) have expressed their support for ASEAN’s intentions to construct smart urbanization in the region. ^71^ Interested actors include Australia, China, India, Japan, and the US, which have already used the EAS platform to exert their influence on the smaller states of Southeast Asia. ^9^ Australia pledged funding for digital solutions concerning ASCN, with the additional financing of the Asian Development Bank. ^72^ Through its Japan International Cooperation Agency, Japan’s know-how has made it a strong destination for cities seeking cooperation from business entities. ^73^ China’s Belt and Road Initiative and a large amount of financial capital have also attracted considerable attention from the ASCN members, who have previously received investments for their country’s strategic development plans. ^37, 74, 75^

Nevertheless, the expansion of the ASCN membership and involvement of business entities have not curtailed the core issue of sustainability concerns with smart cities. The following section will argue that the diverse economic, social, political, and environmental indicators among Southeast Asian states lead to questions and concerns over sustainability within ASEAN’s conceptions of smart cities.

### 3.2 Revisiting the ‘Helix Model’

When the Charles David Crumpton-led ‘quintuple helix model’ article was published, the study made interesting observations. Smart city conceptualizations under the ASCN did not correspond directly to sustainability measures. Therefore, the ideal state is that “urban planning, management, and policy processes must be designed and operated with sustainability objectives in mind and supported by globally recognized dimensions of good governance, including responsiveness, accountability, transparency, and inclusiveness”. ^20^ In alignment with the article’s helix model, the study recommended the situating of smart city conceptualization under a ‘system of systems,’ recognizing “the complex socio-ecological interaction between society and nature and the multi-layered character of governance”. ^20^

The basis of the problem for the ASCN is diversities in systems. The smart city approach becomes complex when applied to governments with authoritarian or semi-authoritarian settings, which could lead to further control over populations. ^76^ For the more democratic nations of Southeast Asia, the problem then becomes to what extent state policymakers consider environmental costs overreaching development targets. It is argued in this section that old patterns are occurring, with the lack of relationship between the citizens and the smart city developments taking place in Southeast Asia. In doing so, the following will explain several indicators that show the diversities between the states and cities in Southeast Asia, indicating a different starting point among the ASCN members, which risks exacerbating the disparities of basic urban services delivered.

Table 1 below shows how diverse Southeast Asian countries are, showing more significant disparity in the past four years. The indicators consider several dimensions that are pivotal in constructing smart cities. First is the 2024 GDP growth, published by the International Monetary Fund, and considers the total value of goods and services produced within the country. ^77^ Second is the 2022 UN Human Development Index (HDI), which considers education, health, and standard of living. ^78^ Third is the 2024 Economist Intelligence Unit’s Democracy Index (EIU), which considers civil liberties, the electoral process, political participation & culture, and the functioning of the government. ^79^ Fourth is the 2024 Reporters Without Borders’ World Press Freedom Index (WPFI), which ranks states based on the assessment of non-government organizations towards freedom of journalists and media. ^80^ Fifth is the 2024 Yale University’s Environmental Performance Index (EPI), which considers the state of sustainability (climate change performance, environmental health, ecosystem vitality). ^81^ Last, which differs from the datasets used in the 2021 study helix model assessment on ASCN, is the 2024 World Bank’s EoDB ranks, which rates the difficulty of starting businesses, issuing construction permits, access to electricity, registering properties, and getting credits, as considerations. ^82^

**Table 1.** Diversities in the political, social, environmental, and economic landscape of Southeast Asian states.

Country	GDP Growth %	HDI/1	HDI global rank	EIU/10	EIU global rank	WPFI/100	WPFI global rank	EPI/100	EPI global rank	EoDB global rank
Brunei	2.4	0,823	55	NA	NA	50.09	117	48.3	69	66
Cambodia	5.5	0,600	148	3.05	121	34.28	151	31.2	169	187
Indonesia	5	0,713	112	6.53	56	51.15	111	33.6	162	73
Lao PDR	4.1	0,620	139	1.71	159	33.76	153	26.3	177	154
Malaysia	4.8	0,807	63	7.29	40	52.07	107	41	118	12
Myanmar	1	0,608	144	0.85	166	24.41	171	27.1	176	165
Philippines	5.8	0,710	113	6.66	53	43.46	134	32.1	168	95
Singapore	2.6	0,949	9	6.18	69	47.19	126	53	47	2
Thailand	2.8	0,803	66	6.35	63	58.12	87	45.7	90	21
Vietnam	6.1	NA	NA	2.62	136	22.31	174	24.6	179	187

Source: UN Development Index (2024), International Monetary Fund (2024), Economist Intelligence Unit (2024), Reporters Without Borders (2023), Environmental Performance Index (2024).

What has changed in the past four years regarding the diversities of the political, social, environmental, and economic landscape of Southeast Asian states? As shown in Table 1 above, the differences are deepened. Discussing the quality of human development would place a country like Singapore in the 9 ^th^ rank globally, but at the same time, Cambodia (which includes Phnom Penh in the ASCN pilot city list) is ranked one of the worst in the world at 148 ^th^.

The EIU and WPFI figures show equally how difficult it is to establish a smart city in Southeast Asia. As seen in Table 1, democracy and the state protection of press freedom is not an easy policy to adopt in the democratic-lacking region of Southeast Asia. On those indicators, the ranks of Myanmar and Vietnam are particularly concerning, ranked among the worst globally. Observers have often highlighted the problem of human rights, democracy, and media press as one of the weaknesses of ASEAN’s institutions. ^40– 42, 83– 85^ With the two low-ranked states in the EIU and WPFI indicators, the fear is that continued repression would take place as the ASCN’s members of Ho Chi Minh City (Vietnam), Hanoi (Vietnam), and Yangon (Myanmar) would be geared to establish the cities as becoming ‘smart’ without the foundational civic and people relations being fixed prior to that development. As a consequence, consistent with the 2021 finding, there is a more substantial possibility of the ASCN being used in less democratic settings to accelerate surveillance and state control.

Meanwhile, the global ranks on EPI and EoDB show some unique developments in Southeast Asian states’ environment and business landscapes. Again, the disparities can be seen in countries like Singapore, which is ranked 47 ^th^ in the EPI, and Vietnam, which is 179 ^th^, showing that ASEAN member states have different environmental priorities. Furthermore, the EoDB figures shown in Table 1 indicate the role of political and government systems in the ease of doing business in ASEAN countries. Singapore is the second best country in the world to do business, but its ASEAN counterpart, Cambodia, is ranked 187 ^th^, one of the lowest in the world.

How about the diversity of the cities of ASCN? Table 2 below provides an update of the recent ranks of cities based on the 2024 Globalization and World Cities figures, ranking cities based on the assessment of 175 leading firms producing transnational services. ^86^ The problem with recent findings is that half of the members of the ASCN are not even considered cities that strongly impact global services. However, several countries are ranked among the most significant cities in the world, including Singapore, Jakarta, and Kuala Lumpur. Several completed and ongoing ASCN projects have been implemented in those cities, with programs geared to solidifying the importance of those cities through technological advancement in the public services sector.

**Table 2.** Rank of the importance of cities, based on the assessment of the Globalization and World Cities.

GaWC 2024 categorization	ASCN City	Completed and Ongoing ASCN Smart City Projects (as of September 2024)
Alpha+	Singapore	E-Payments (completed), National Digital Identity (completed), Smart Nation 1.0 Initiatives (completed), Punggol Digital District (ongoing), Woodlands North Coast (ongoing)
Alpha	Jakarta, Indonesia	JakPreneur (ongoing), JakLingko (ongoing), JAKI (ongoing), Jakarta Smart City Goes to School (ongoing)
Alpha	Kuala Lumpur, Malaysia	Kuala Lumpur Urban Observatory (ongoing), OSC 3.0 Plus Online (ongoing), GoKL Journey Planner (ongoing), Smart Bin (ongoing), Bicycle Friendly City (ongoing)
Alpha	Bangkok, Thailand	Bang Sue Smart City (ongoing), Bang Sue Grand Station (ongoing)
Beta+	Ho Chi Minh City, Vietnam	Intelligent Operation Centre (ongoing), Integrated and Unified Emergency Response Centre (ongoing)
Beta	Hanoi, Vietnam	Intelligent Operation Centre (ongoing), Transport Operation and Surveillance Centre (ongoing)
Beta	Manila, Philippines	Command Center Upgrade (ongoing), E-Government Services (ongoing)
Gamma	Phnom Penh, Cambodia	Smart City Strategic Planning (completed), Phnom Penh Walk Way (ongoing), Phnom Penh Public Bus Service (ongoing), Phnom Penh Smart City Hub (ongoing), Build4People (ongoing)
Sufficiency	Cebu City, Philippines	Cebu City Bus Rapid Transit (ongoing), Automated Citywide Traffic Control System (ongoing)
Sufficiency	Johor Bahru, Malaysia	Iskandar Malaysia Integrated Urban Services Program (completed), Iskandar Malaysia Analytics Centre (ongoing), Management of Water Resources and Distribution (ongoing), Integrated Smart Mobility Programs (ongoing)
Sufficiency	Vientiane, Laos	Smart Environment Development (ongoing), Vientiane Sustainable Urban Transport (ongoing)
Sufficiency	Yangon, Myanmar	Conservation of Downtown Yangon (ongoing), Transit Oriented Development in Hlaing Thar Yar Township (ongoing), One Map Yangon (ongoing)

Source: ASEAN Smart Cities Network (2024), Globalization and World Cities (2024).

However, what is puzzling is the rising significance of cities in less democratic settings such as Hanoi, Ho Chi Minh City, Phnom Penh, Vientiane, and Myanmar. In the past four years, these four cities have struggled to ensure greater civic rights are maintained. As the ASCN is focused on accelerating the efforts to make these cities ‘smart,’ there seems to be a profound neglect of the political issues in those cities due to this dominant discourse of ‘smart’ development. This raises a substantial concern, as these cities would only focus on consistency with the ASCN smart city framework and further neglect the fundamental urban services.

The ASCN does not consider these disparities in capacities. The focus has been on accelerating the ‘smart city’ development of the pilot city projects of the ASCN and utilizing AI and technology to provide better public services for the city’s population. However, the complexity of this is how about cities in countries that lack democratic systems, show less appreciation towards the environment, and lack proper prospects for human development? The findings of the updated data for the 2021 study show that the same conclusions have been attained. The disparities will only lead to more significant differences among the ASCN members, with the absence of sustainability measures with the ASCN projects.

Therefore, the ‘smart city’ of ASCN may not be the best development course for Southeast Asian states. Past studies have noted that the characteristics of smart cities tend to be referenced in contemporary urban planning. ^87– 89^ The ‘smart city’ discourse severely underestimates the long process it takes for cities to develop and views that cities can simply be ‘updated’ just like programs and technologies. ^90, 91^ The ASCN does not discriminate between the diversities of ASEAN member states and, therefore, does not differentiate based on the needs of more extensive, smaller, democratic, or authoritarian settings.

The problem with diverse landscapes such as ASEAN is that everything ‘smart’ may easily lead to deepening authoritarian tendencies and work against sustainability objectives. ^20^ Another issue is the lack of acknowledgment that the starting points of these cities are different, with some already building the foundations of smart cities since the 1960s. ^90^ As a consequence, it is expected after observing recent data in the past four years that poor cities would most likely see a more substantial deterioration of sustainability norms, as anticipated in past studies. ^92– 94^

## 4. Conclusion

The ‘quintuple helix model’ provided the basis for assessing the nexus between the ASCN and sustainability. Interactions among actors within the state are what establish and boost innovation systems, which are essential in generating balanced approaches in development. The 2021 study concluded that the vast disparities and differences among Southeast Asian states had severely impeded efforts in sustainable urban planning and governance, affecting how the ASCN is able to balance development interests with sustainable measures. The aim of this study is to build up on the past narrative, updating data on the political, social, environmental, and economic landscape of Southeast Asian states between 2021 and 2024, severely affecting how the region’s ASCN participating cities are able to incorporate sustainable measures. In terms of the emphasis on economic disparity, additional data on the EoDB among ASEAN member states is also considered.

As predicted, smart city approaches are challenging to adopt in a region containing authoritarian or semi-authoritarian settings. Consistent with the conclusions of the past study, this updated research finds a lack of good governance, accountability, inclusiveness, and transparency in many of the participating ASCN members. Consequently, no direct correlation is seen between achieving the smart city status and sustainability. As seen with the EIU and WPFI figures presented in this study, democracy and state protection of press freedom are still lacking in many of the Southeast Asian states. The concern, therefore, is that cities such as Ho Chi Minh City, Hanoi, and Yangon are geared toward achieving smart city status despite lacking the foundational civic and people relations, raising concerns over the trajectory of its cities’ developments. The disparity among ASEAN member states is also seen in the global ranks of the EPI and EoDB, with some countries, such as Singapore, ranked among the highest globally, while others, such as Cambodia, ranked in one of the lowest in terms of environment and business landscapes. Unfortunately, the issue found at the state level among the ASEAN member states applies to its participating ASCN cities. The case is clear with Hanoi, Ho Chi Minh City, Phnom Penh, Vientiane, and Myanmar, which, based on the Globalization and World Cities ranking, have struggled to show greater care for civic rights. Therefore, rather than focusing on fixing such profound neglect of political rights, the focus, as instructed in the ASCN, has been to adopt smart city measures. The fear would be that the cities neglect fundamental urban services.

The findings of this study extend the existing discourse on the lack of connection between smart city development programs and sustainability. The term smart city is a common term referenced in contemporary urban planning. However, this severely undermines the long process a city needs to develop. It is as if cities can simply be updated with state-of-the-art technologies. In the case of the ASCN participating cities, the concern is the disparity in capacity, lack of fundamental civic and human rights, and different problems within the cities. These disparities lead to different participation, governance, and socio-ecological sustainable planning, which are not precisely captured under the smart city concept of the ASCN. In the case of ASCN cities residing in authoritarian and semi-authoritarian settings, there is a tendency for authoritarian systems to deepen amid calls for greater surveillance systems and movement control from the central government. Seeing the recent progress taking place in the Southeast Asian region, a deterioration of sustainability norms is observed, and this will likely continue if the primary narrative introduced as a means for city-based developments is smart city conceptions.

## Declaration of the use of AI

No Generative AI or AI-assisted technologies were used in the writing of this manuscript.

## Data Availability

The data that support the findings of this study can be accessed below:
1. World Economic Outlook 2024 (IMF): https://www.imf.org/en/publications/weo/issues/2024/10/22/world-economic-outlook-october-2024
2. Human Development Index 2022 (UNDP): https://hdr.undp.org/data-center/human-development-index#/indicies/HDI
3. Democracy Index 2024 (Economist Intelligence): https://www.eiu.com/n/campaigns/democracy-index-2024/
4. World Press Freedom 2024 (Reporters Without Borders): https://rsf.org/en/2024-world-press-freedom-index-journalism-under-political-pressure
5. Environmental Performance Index 2024 (Yale University): https://epi.yale.edu/measure/2024/epi
6. Ease of Doing Business 2024 (World Bank): https://archive.doingbusiness.org/en/rankings
7. World Cities 2024 (Globalization and World Cities): https://gawc.lboro.ac.uk/gawc-worlds/the-world-according-to-gawc/world-cities-2024/
