# Are Smart Cities ‘Sustainable?’ Revisiting the ‘Helix Model’ of the ASEAN Smart Cities Network

**DOI:** 10.12688/f1000research.174616.2

**Published:** 2026-04-24

**Authors:** Bama Andika Putra

**Affiliations:** 1 International Relations, Hasanuddin University Faculty of Social and Political Sciences, Makassar, South Sulawesi, Indonesia; 2 University of Bristol School of Sociology Politics and International Studies, Bristol, England, UK

**Keywords:** Smart Cities, ASEAN, Helix Model, Sustainability, Southeast Asia

## Abstract

Recent urban planning in Southeast Asia has incorporated the theme of transforming cities to ‘smart city’ status. However, the central question raised in this study is whether the ASEAN’s conception of smart cities is genuinely sustainable. Based on the backdrop of Southeast Asia’s disparity in economic performances and diversity in its political, social, economic, and environmental dimensions, this study updates Crumpton’s 2021 study on the ‘quintuple helix model’ and argues that new observations but old patterns are found in the ASEAN Smart Cities Network’s way of developing cities in Southeast Asia. Through a qualitative document analysis, utilizing published secondary data on the ten ASEAN member states between 2020 and 2024, this study revisits and updates the current state of Southeast Asia and how this corresponds to the challenges of constructing smart cities in democratic, semi-authoritarian, and authoritarian settings.

## 1. Introduction

Are smart cities sustainable? Past studies have questioned whether smart city approaches adopted by local governments lead to urban sustainability. ^1^^–^^5^ In recent years, we have seen cities adopt smart-city grand-strategy schemes to accelerate growth. Nevertheless, how many compromises are made to meet current and future needs?

Among the regions that have actively adopted this smart city concept is Southeast Asia. Under the Association of Southeast Asian Nations (ASEAN), particularly during Singapore’s ASEAN chairmanship in 2018, member states agreed with a list of 26 pilot projects under the ASEAN Smart Cities Network (ASCN). ^6^ The growth of cities in ASEAN is unique, as they will “continue to be driven by urban centers, with more people expected to urbanize by 2030 and ‘middleweight’ cities of between 200,000 and 2 million residents forecast to drive 40% of the region’s growth”. ^7^ The role of ASCN is thus to act as a normative guideline for ASEAN member states on smart city development, sharing best practices, and linking partnerships to accelerate growth. ^6^^,^^8^ The expectation is that the ASCN will act as a facilitator, linking city government officials and business entities to “catalyze bankable projects to solve urban problems by utilizing technology and innovation opportunities provided by the private sector”. ^9^

For ASEAN, the strategic outcomes of smart cities consist of a high quality of life, a competitive economy, and a sustainable environment. ^6^ To achieve them, several development focus areas have been determined: civic and social, health and well-being, safety and security, quality environment, built infrastructure, and industry and innovations. The identified development areas are enabled by ASEAN’s encouragement of digital infrastructure and applications, as well as by partnership and funding schemes under the ASCN. ^10^

Scholars have interpreted the ASCN in different ways. The most common discourse introduced is that of local governments’ active participation in offering and seeking partnerships as a representation of ‘paradiplomacy’, as non-central government actors engage in international relations. ^7^^,^^9^^,^^11^^,^^12^ The interlinkages between cities and business entities have also led some studies to conclude that the ASCN exhibits ‘transboundary learning’. ^13^^,^^14^ Nevertheless, the more recent discourse aims to connect sustainability with the smart city conceptions of the ASCN, with studies focusing on the impacts on tourism, human rights, and governance. ^12^^,^^15^^,^^16^

This article seeks to update Charles David Crumpton’s ‘quintuple helix model’ to assess the nexus between the ASCN and sustainability. The approach is grounded in the ‘helix models’ that symbolize the interactions among different sectors. ^17^^,^^18^ The interactions considered include actors in the education, entrepreneurial, public, media, and ecological contexts, ^19^^–^^23^ which collectively can boost regional innovation systems. The Crumpton-led 2021 study concluded that the diversity found in Southeast Asian states, some of which have authoritarian systems, has severely impeded the “socio-ecologically sustainable urban planning and governance” of the ASCN’s smart cities. ^20^

In the ASCN’s development focus area of civic and social, for example, some countries, such as Vietnam, Laos, Cambodia, and Brunei Darussalam, still lack the basic features of democracy. ^24^^–^^26^ Thus, the intention of its cities to transform into smart cities is seen as a potential policy to further impinge upon participatory and socio-ecological sustainable urban planning and governance. ^20^ Therefore, as seen in the case of the ASCN in recent years, Crumpton highlights the emergence of the state under-prioritizing the environment and the lack of initiatives in improving basic services. This inadequate consideration of inter-contextual differences among ASEAN member states means that members would struggle to achieve smart city status, due to the underlying issue of unmet urban service requirements.

Through a qualitative document analysis, this study intends to provide an update on the ASCN under the ‘helix models.’ It considers several unique trends between 2020 and 2024 that contribute to the diversity of Southeast Asian states. These include the Myanmar military coup in 2021, Laos’ foreign debt surges, and Cambodia’s change of leadership after more than three decades, to name a few. ^27^^–^^33^

Recent developments since 2021 have revealed a more complex Southeast Asia, making it difficult to introduce sustainability measures. In its current form, most Southeast Asian states are sensitive to the notion of imposed regulations and systems, as the region is founded on the norm of non-interference. ^34^^,^^35^ Consequently, through ASEAN, Southeast Asian states agree that introducing new norms into the region will be undertaken at a slow pace, one that accommodates the region’s unique differences. In addition, ASEAN’s consensus-based decision-making system allows the organization to adopt mechanisms and regulations only if no member state objects. Some unique trends further exacerbate such a unique context in Southeast Asia’s politics. In many parts of Southeast Asia, there is a more apparent dependence on China’s Belt and Road Initiative (BRI), which, unfortunately, has led to an increase in foreign debt for smaller Southeast Asian states. ^36^^–^^39^ Democracy is another prominent problem in the region. With Myanmar’s undemocratic rule and questions over Cambodia’s stability following leadership changes, there is doubt about whether Southeast Asia can put aside differences to pursue sustainability measures. In the past, pressing domestic challenges and a lack of democracy also contributed to the region’s failure to take decisive measures to protect human rights at the ASEAN level. ^40^^–^^42^

Consequently, these changes within the political landscape of Southeast Asian states make the inquiry of sustainability in smart cities particularly interesting to re-explore. Inspired by Crumpton’s 2021 analysis, this study will provide the ‘helix model’s’ interpretation of the ASCN, with the following differences. First, the inclusion of more recent data (2020–2024) accounts for differences in the political, social, environmental, and economic landscapes of Southeast Asian states. Second is the exclusion of one of the proposed models of interactions among educational stakeholders, due to the inconclusive relationship between information and communication technology projects and high-ranked universities. ^20^ Third is the inclusion of the Ease of Doing Business (EoDB) rankings to complement past data on the differences in the Southeast Asian economic landscape. The intention of this study is thus similar to that of past studies, which aim to reveal the profoundly diverse region of Southeast Asia and the challenges it poses to implementing sustainability measures within the ASCN’s conception of smart cities.

## 2. The ASEAN, ASCN, Smart Cities, and Sustainability: A Literature Review

Against the backdrop of unique norms and differences among states, adopting sustainability measures in Southeast Asia poses challenges. The existing literature highlights these challenges well, focusing on the trajectory of sustainability among ASEAN member states over the past years. Nevertheless, not enough attention has been paid to ASEAN’s development pathway, especially given its recent emphasis on advancing it through the construction of smart cities across the region. This section provides a brief literature review of how Southeast Asia engages with sustainability demands. First, it examines the challenges of adopting sustainability across different regional sectors and the tendency in the literature to call for modified governance and policies to address these concerns. Second, this section examines how the ASCN has been one of ASEAN’s solutions to address urbanization issues in Southeast Asia’s larger cities, focusing on smart city initiatives as the basis for these solutions. Lastly, it will revisit recent studies on smart cities and sustainability in Southeast Asia and the novelty this study offers. These three discourses allow for a more nuanced understanding of the dominant discussions on smart cities and sustainability in Southeast Asia, and of the importance of revisiting more recent empirical case studies to strengthen or challenge past interpretations.

### 2.1 Sustainability in Southeast Asia: Conflicting Norms?

Perhaps the primary discourse relevant to this study is studies exploring the sustainability element of Southeast Asian states’ practices across time. Within this discourse, studies have been particularly critical in questioning whether Southeast Asian states have ensured sustainable measures are in place concerning the vast development projects. Based on evaluations across sectors, scholars agree that Southeast Asia still faces challenges in integrating sustainability measures into its development practices. Ruland, for example, argued that path dependencies and development practices are the reason Southeast Asia’s hydropower dams lack elements of environmental sustainability. ^43^ Also, in the energy sector, scholars have looked into how carbon emissions in Southeast Asian states have faced an increase in volume due to the region’s focus on economic development and the encountered challenges of inadequate infrastructure and limitations of technologies. ^44^ Linked to the field of tourism, other studies have pointed to Southeast Asia’s tourism policies as lacking consideration of potential environmental elements, which consequently leads to unsustainable practices. ^45^ As Putra rightfully concludes when assessing the politics of countering climate change in the region, Southeast Asia’s unique norms of non-interference and persistence in undertaking development practices to advance their respective nations have equally contributed to the lack of sustainability practices in the region. ^46^

Although the highlight of challenges has been the dominant discourse, some studies have examined what the ideal solution could be. Studies have argued that policymakers should re-evaluate their good governance to ensure environmental sustainability. ^47^ Others have argued for the nexus between corporate governance and corporate sustainability disclosures, asserting that it serves as a baseline for ensuring that development in the region is balanced with careful consideration of the environment. ^48^ However, other studies have also pointed out that the issue is multidimensional and needs simultaneous approaches. Dai, for example, in a recently published article, called for the following measures: tax incentives, financial institution collaborations, ESG investing, and transparency. ^49^

Another discourse worth mentioning is the development gap between ASEAN member states. The challenges and potential solutions outlined in past studies are common problems all states face. Nevertheless, finding a balanced solution for ASEAN has been challenging for its member states due to disparities in capacity among Southeast Asian states. Past studies have highlighted the problem well. ^50^^–^^52^ Most have looked into the newer ASEAN states, often referred to as the CMLV (Cambodia, Myanmar, Laos, Vietnam), as these states still lack resources and supporting systems to pursue a balanced development approach. ^53^ Consequently, sustainability is enhanced when discussing such states. Nevertheless, no direct connections are made to how, for example, the lack of democratic practices in those states (or any Southeast Asian state) would impact their sustainability practices. As part of this solution, ASEAN established the ASCN, focusing on city actors as the basis of its solution. How has this been interpreted in existing studies?

### 2.2 Is the ASCN the Solution to the Slow Performing Sustainability Measures in the Region?

Given the challenges of development and sustainability across much of Southeast Asia, the ASCN has been one of ASEAN’s solutions. One reason this has surfaced is urbanization-related challenges, with cities as the primary stakeholders who can make direct changes. Arfanuzzaman and Dahiya, for example, argue that urbanization in the region is uncontrollable due to its haphazard nature. ^54^ A study in 2020 and 2021 found that the urbanization challenges faced in Asia mimic those of Western cities, with the larger cities of Southeast Asia, such as Kuala Lumpur, Jakarta, and Manila, encountering the challenge of population densities. ^55^^,^^56^ These more recent studies are consistent with the earlier findings of Giok Ling Ooi in 2008 and 2009. Her studies predicted that Southeast Asian cities would experience in-migration and urban sprawl, leading to long-term environmental degradation. ^57^ In her 2009 study, she argued that such states fail to “[…] identify and implement an urban development policy framework that will link the effort at more sustainable development in a variety of sectors”. ^58^

Nevertheless, the city-oriented solution is not solely due to urbanization. Studies have examined decentralization as a primary factor influencing urban resilience. Marks and Pulliat looked into how fragmented governance structures and “misaligned incentive structures” are causing uneven commitment among cities to tackle climate change. ^59^ Amid the rise of such challenges, scholars have looked into different conceptions to alleviate the impact of the lack of sustainability measures adopted within Southeast Asian states. These include an inquiry into the potential for smart cities to be adopted in the region and into the development of smart cities as a foundation for sustainable cities. ^15^^,^^60^

Established in 2018, the ASCN has quickly attracted scholarly attention, prompting questions about whether it would contribute to the development of more sustainable cities. Most studies have concluded in favor of the initiative, arguing that the ASCN would catalyze partnerships and support the region’s digitization efforts. ^9^^,^^61^ Others have looked at the advantages of the ASCN due to its potential to establish transboundary learning ^14^ and “[…] fostering autonomy-enhancing initiatives between developing countries that have the capacity to learn from and scale-up locally-informed, adaptive problem solving”. ^16^ Nevertheless, some studies have been critical in addressing the ASCN. Two concerns have been prominent: First is the impact of ‘technocratic regionalism,’ defined as the technologically-driven regional integration resulting from the ASCN, ^62^ and second, how achieving the ‘smart city’ status would not be able to be balanced with human rights concerns that have already become a significant challenge for many Southeast Asian states. ^12^ Based on that backdrop, it is clear that the smart city destination the ASCN aims to achieve for its participating cities can yield both positive and negative outcomes. The following subsection will examine how past studies have interpreted this dilemma and elaborate on the novelty of this updated study.

### 2.3 Revisiting Discussions on Smart Cities and Sustainability in Southeast Asia

Studies in the past have raised doubts about whether smart cities are indeed sustainable means of developing cities. As past studies have shown, there is unclear evidence that shows that the smart city approach indeed leads to urban sustainability. ^1^^,^^3^^,^^5^^,^^63^^,^^64^ In building up such an assumption, Charles David Crumpton, Supawatanarkorn Wongthanavasau, Peerasit Kamnuansilpa, John Draper, and Eva Bialobrzeski specifically analyzed this in the context of the ASCN. Adopting the ‘helix models,’ the study looks into specific city plans in Southeast Asia, the ASCN, and experiences with the smart city initiative. ^20^ The study highlights the disparities in social, economic, and political rights among Southeast Asian states and argues that the presence of states with authoritarian settings generates a more significant challenge for the ASCN. It is then proposed that the smart city conceptualization be within a ‘system of systems’ framework. ^20^ In this, “the complex socio-ecological interaction between society and nature and the multi-layered character of governance are acknowledged”. ^20^

Consequently, the disparity in economic capacity, social and human rights, and political systems between the Southeast Asian states is perceived as a barrier to achieving the ideal smart city status for ASCN participating cities. With the technologies proposed as the basis for a smart city, ASEAN is raising the potential for abuse and heightened cyber surveillance, which could be the case in many authoritarian nations in the region. As Crumpton’s-led study mentioned, “[…] authoritarian regimes and regimes with authoritarian tendencies seek new and better means for surveilling and controlling their populations”. ^20^ And that such state systems would impact “[…] participatory and therefore socio-ecologically sustainable urban planning and governance”. ^20^

This study builds on the previous concerns on the nexus between smart cities and sustainability in Southeast Asia. Between 2020 and 2024, many developments have contributed to capacity disparities, political tensions, and infringements on social and human rights. Unfortunately, to balance development and sustainability, ASEAN has focused on the ASCN as one of the primary means of achieving balanced development for the region’s larger cities. The novelty of this study thus offers the opportunity to reveal the profound problems associated with the ASCN, which distances itself from actual sustainability measures as its primary aim and further impacts participatory and socio-ecological sustainable urban planning and governance. In doing so, it follows the 2019 framework and highlights how the region’s socio-political developments have contributed to the disparities.

## 3. The ‘Helix Model’ of the ASCN: New Observations, Old Patterns

### 3.1 Recent Developments of the ASCN

The original plan of the ASCN is threefold: First, it will facilitate cooperation on smart city development, catalyze bankable projects, and secure funding from ASEAN’s external partners. ^65^ Established during Singapore’s ASEAN chairmanship in 2018, ASEAN envisioned that, despite differences in political systems and economic landscapes among the member states, the vision to develop smart cities within Southeast Asia would be perceived as a priority for local governments. As of September 2024, the total number of ASCN’s smart city projects accumulates to 108. ^6^ Divided into the development areas of the ASCN, 27% of projects are civic and social, 18% on quality environment, 6% on health and well-being, 26% on built infrastructure, 12% on safety and security, and 11% on industry and innovation. ^6^ At the core of facilitating smart city development, the ASCN Smart Cities Framework continues to articulate the essential features that ASEAN cities aim to achieve.

Cities incorporated into the ASCN have expanded. The pilot ASCN cities initially incorporated 26 cities from the ten ASEAN members. Since June 2024, the list has grown to include five new cities, including Chiang Mai (Thailand), Khon Kaen (Thailand), Rayong (Thailand), Sumedang (Indonesia), and Sihanoukville (Cambodia). ^10^ Already in this list of ASCN are notable Southeast Asian capital cities such as Jakarta, Bangkok, Manila, Kuala Lumpur, and Singapore. These cities have seen rapid urbanization, with common issues in their public services. ^66^^–^^69^ The challenges of urbanization, employment opportunities, environmental degradation, people’s welfare, and inadequate transportation facilities are common problems across the pilot ASCN cities. ^9^^,^^12^^,^^16^^,^^70^

In recent years, Southeast Asian cities have actively engaged in paradiplomacy to secure funding from ASEAN’s external partners. Since 2018, members of the East Asia Summit (EAS) have expressed their support for ASEAN’s intentions to construct smart urbanization in the region. ^71^ Interested actors include Australia, China, India, Japan, and the US, which have already used the EAS platform to exert their influence on the smaller states of Southeast Asia. ^9^ Australia pledged funding for digital solutions related to ASCN, with additional financing from the Asian Development Bank. ^72^ Through its Japan International Cooperation Agency, Japan’s know-how has made it a strong destination for cities seeking cooperation from business entities. ^73^ China’s Belt and Road Initiative and a large amount of financial capital have also attracted considerable attention from the ASCN members, who have previously received investments for their country’s strategic development plans. ^37^^,^^74^^,^^75^

Nevertheless, the expansion of ASCN membership and the involvement of business entities have not addressed the core sustainability concerns of smart cities. The following section will argue that the diverse economic, social, political, and environmental indicators across Southeast Asian states raise questions and concerns about sustainability within ASEAN’s conceptions of smart cities.

### 3.2 Revisiting the ‘Helix Model’

When the Charles David Crumpton-led ‘quintuple helix model’ article was published, the study made interesting observations. Smart city conceptualizations under the ASCN did not correspond directly to sustainability measures. Therefore, the ideal state is that “urban planning, management, and policy processes must be designed and operated with sustainability objectives in mind and supported by globally recognized dimensions of good governance, including responsiveness, accountability, transparency, and inclusiveness”. ^20^ In alignment with the article’s helix model, the study recommended the situating of smart city conceptualization under a ‘system of systems,’ recognizing “the complex socio-ecological interaction between society and nature and the multi-layered character of governance”. ^20^

The basis of the ASCN’s problem is diversity across systems. The smart city approach becomes complex when applied to governments with authoritarian or semi-authoritarian settings, which could lead to further control over populations. ^76^ For the more democratic nations of Southeast Asia, the problem then becomes to what extent state policymakers consider environmental costs as exceeding development targets. This section argues that old patterns are emerging, with a lack of relationship between citizens and the smart city developments taking place in Southeast Asia. In doing so, the following explains several indicators that show the diversity between states and cities in Southeast Asia, highlighting different starting points among the ASCN members, which risks exacerbating disparities in the delivery of basic urban services. Similar to Crumpton’s 2021 point, the updated empirical data presented in the two tables below from 2020 to 2024 show that concern over non-democratic practices can negatively affect urban planning and governance, underplay environmental concerns, and neglect improvements in the quality of life of the city’s population.

Table 1 below shows how diverse Southeast Asian countries are, with a more significant disparity in recent years. Although the presented data pertain to state actors, there is a strong likelihood that the patterns of democracy, GDP growth, human development, press freedom, ease of doing business, and environmental performance at the state level are also of concern to local and regional governments. This suggests that half of Southeast Asian states are non-democratic, indicating that a hierarchical system will govern the concerns government officials focus on. The indicators consider several dimensions pivotal to the construction of smart cities. First is the 2024 GDP growth, published by the International Monetary Fund, which considers the total value of goods and services produced within the country. ^77^ Second is the 2022 UN Human Development Index (HDI), which considers education, health, and standard of living. ^78^ Third is the 2024 Economist Intelligence Unit’s Democracy Index (EIU), which considers civil liberties, the electoral process, political participation & culture, and the functioning of the government. ^79^ Fourth is the 2024 Reporters Without Borders’ World Press Freedom Index (WPFI), which ranks states based on assessments by non-governmental organizations of journalists’ and media freedom. ^80^ Fifth is the 2024 Yale University’s Environmental Performance Index (EPI), which considers the state of sustainability (climate change performance, environmental health, ecosystem vitality). ^81^ Last, which differs from the datasets used in the 2021 study helix model assessment on ASCN, is the 2020 World Bank’s EoDB ranks, which rate the difficulty of starting a business, obtaining construction permits, accessing electricity, registering property, and obtaining credit. ^82^

What has changed in the past four years regarding the diversities of the political, social, environmental, and economic landscape of Southeast Asian states? As shown in Table 1 below, the differences are deepened. Discussing the quality of human development would place a country like Singapore in the 9 ^th^ rank globally, but at the same time, Cambodia (which includes Phnom Penh on the ASCN pilot city list) is ranked among the worst in the world at 148 ^th^.

**Table 1.** Diversities in the political, social, environmental, and economic landscape of Southeast Asian states.

Country	GDP Growth %	HDI/1	HDI global rank	EIU/10	EIU global rank	WPFI/100	WPFI global rank	EPI/100	EPI global rank	EoDB global rank
Brunei	2.4	0,823	55	NA	NA	50.09	117	48.3	69	66
Cambodia	5.5	0,600	148	3.05	121	34.28	151	31.2	169	144
Indonesia	5	0,713	112	6.53	56	51.15	111	33.6	162	73
Lao PDR	4.1	0,620	139	1.71	159	33.76	153	26.3	177	154
Malaysia	4.8	0,807	63	7.29	40	52.07	107	41	118	12
Myanmar	1	0,608	144	0.85	166	24.41	171	27.1	176	165
Philippines	5.8	0,710	113	6.66	53	43.46	134	32.1	168	95
Singapore	2.6	0,949	9	6.18	69	47.19	126	53	47	2
Thailand	2.8	0,803	66	6.35	63	58.12	87	45.7	90	21
Vietnam	6.1	0,726	107	2.62	136	22.31	174	24.6	179	70

Source: UN Development Index (2024), International Monetary Fund (2024), Economist Intelligence Unit (2024), Reporters Without Borders (2023), Environmental Performance Index (2024).

The EIU and WPFI figures show that it is equally difficult to establish a smart city in Southeast Asia. As seen in Table 1, democracy and state protection of press freedom are not easy to implement in the democratic-deficient region of Southeast Asia. On those indicators, Myanmar and Vietnam rank among the worst globally. Observers have often highlighted the problems of human rights, democracy, and the media press as among the weaknesses of ASEAN’s institutions. ^40^^–^^42^^,^^83^^–^^85^ With the two low-ranked states in the EIU and WPFI indicators, the fear is that continued repression would take place as the ASCN’s members of Ho Chi Minh City (Vietnam), Hanoi (Vietnam), and Yangon (Myanmar) would be geared to establish the cities as becoming ‘smart’ without the foundational civic and people relations being fixed before that development. As a consequence, consistent with the 2021 finding, there is a greater likelihood that the ASCN will be used in less democratic settings to accelerate surveillance and state control.

Meanwhile, the global rankings on EPI and EoDB reveal unique developments in Southeast Asian states’ environmental and business landscapes. Again, the disparities can be seen in countries like Singapore, ranked 47 ^th^ in the EPI, and Vietnam, ranked 179 ^th^, showing that ASEAN member states have different environmental priorities. Furthermore, the EoDB figures shown in Table 1 indicate the role of political and government systems in the ease of doing business in ASEAN countries. Singapore is the second-best country in the world to do business, but its ASEAN counterpart, Cambodia, is ranked 144 ^th^, among the lowest.

How about the diversity of the cities of ASCN? Table 2 below provides an update of the recent ranks of cities based on the 2024 Globalization and World Cities figures, ranking cities based on the assessment of 175 leading firms producing transnational services. ^86^ The problem with recent findings is that half of the members of the ASCN are not even considered cities that strongly impact global services. However, several countries are ranked among the most significant cities in the world, including Singapore, Jakarta, and Kuala Lumpur. Several completed and ongoing ASCN projects have been implemented in those cities, with programs geared to solidifying the importance of those cities through technological advancement in the public services sector.

**Table 2.** Rank of the importance of cities, based on the assessment of the Globalization and World Cities.

GaWC 2024 categorization	ASCN City	Completed and Ongoing ASCN Smart City Projects (as of September 2024)
Alpha+	Singapore	E-Payments (completed), National Digital Identity (completed), Smart Nation 1.0 Initiatives (completed), Punggol Digital District (ongoing), Woodlands North Coast (ongoing)
Alpha	Jakarta, Indonesia	JakPreneur (ongoing), JakLingko (ongoing), JAKI (ongoing), Jakarta Smart City Goes to School (ongoing)
Alpha	Kuala Lumpur, Malaysia	Kuala Lumpur Urban Observatory (ongoing), OSC 3.0 Plus Online (ongoing), GoKL Journey Planner (ongoing), Smart Bin (ongoing), Bicycle Friendly City (ongoing)
Alpha	Bangkok, Thailand	Bang Sue Smart City (ongoing), Bang Sue Grand Station (ongoing)
Beta+	Ho Chi Minh City, Vietnam	Intelligent Operation Centre (ongoing), Integrated and Unified Emergency Response Centre (ongoing)
Beta	Hanoi, Vietnam	Intelligent Operation Centre (ongoing), Transport Operation and Surveillance Centre (ongoing)
Beta	Manila, Philippines	Command Center Upgrade (ongoing), E-Government Services (ongoing)
Gamma	Phnom Penh, Cambodia	Smart City Strategic Planning (completed), Phnom Penh Walk Way (ongoing), Phnom Penh Public Bus Service (ongoing), Phnom Penh Smart City Hub (ongoing), Build4People (ongoing)
Sufficiency	Cebu City, Philippines	Cebu City Bus Rapid Transit (ongoing), Automated Citywide Traffic Control System (ongoing)
Sufficiency	Johor Bahru, Malaysia	Iskandar Malaysia Integrated Urban Services Program (completed), Iskandar Malaysia Analytics Centre (ongoing), Management of Water Resources and Distribution (ongoing), Integrated Smart Mobility Programs (ongoing)
Sufficiency	Vientiane, Laos	Smart Environment Development (ongoing), Vientiane Sustainable Urban Transport (ongoing)
Sufficiency	Yangon, Myanmar	Conservation of Downtown Yangon (ongoing), Transit Oriented Development in Hlaing Thar Yar Township (ongoing), One Map Yangon (ongoing)

Source: ASEAN Smart Cities Network (2024), Globalization and World Cities (2024).

However, what is puzzling is the rising significance of cities in less democratic settings such as Hanoi, Ho Chi Minh City, Phnom Penh, Vientiane, and Myanmar. In the past four years, these four cities have struggled to ensure greater civic rights are maintained. As the ASCN is focused on accelerating the efforts to make these cities ‘smart,’ there seems to be a profound neglect of the political issues in those cities due to this dominant discourse of ‘smart’ development. This raises a substantial concern, as these cities would focus solely on consistency with the ASCN smart city framework, thereby further neglecting fundamental urban services. In Vientiane and other large cities in Laos, the concern remains the restriction of civil and political rights, including cases of unfair criminal trials and judicial corruption. Meanwhile, in Jakarta, opponents of democratic rule have been met with harsh measures to suppress negative sentiment toward the president. These cases go to show that accelerating development that leans towards being ‘smart’ does not automatically translate into the provision of basic political and human rights services. It also shows a strong linkage between state-level political concerns and local-level appreciation towards human rights.

The ASCN does not consider these capacity disparities. The focus has been on accelerating the ‘smart city’ development of the ASCN’s pilot city projects and on leveraging AI and technology to deliver better public services to the city’s population. However, the complexity is that cities in countries without democratic systems show less appreciation for the environment and lack proper prospects for human development. The updated data from the 2020 study show that the same conclusions have been reached. The disparities will only lead to greater differences among ASCN members, given the absence of sustainability measures in ASCN projects. Perhaps two case studies of concern are in Yangon and Phnom Penh. In Yangon, prosecutions continue amid the military junta’s crackdown on democratic voices. Meanwhile, despite the acceleration of development in Phnom Penh, this has been met with an increase in money laundering, concerns over foreign debt, and rising unemployment, indicating that smart cities’ focus on transportation services remains unbalanced with other essential services.

Therefore, the ‘smart city’ of ASCN may not be the best development course for Southeast Asian states. Past studies have noted that the characteristics of smart cities are often referenced in contemporary urban planning. ^87^^–^^89^ The ‘smart city’ discourse severely underestimates the long process it takes for cities to develop and views cities as if they could be ‘updated’ just like programs and technologies. ^90^^,^^91^ The ASCN does not discriminate among the diversities of ASEAN member states and therefore does not differentiate based on the needs of more extensive, smaller, democratic, or authoritarian settings.

The problem with diverse landscapes such as ASEAN is that everything ‘smart’ can easily deepen authoritarian tendencies and undermine sustainability objectives. ^20^ Another issue is the lack of acknowledgment that the starting points of these cities are different, with some already building the foundations of smart cities since the 1960s. ^90^ As a consequence, it is expected that, after observing recent data in the past four years, poor cities would most likely see a more substantial deterioration of sustainability norms, as anticipated in past studies. ^92^^–^^94^ The case of Hanoi, Vietnam, is interesting given the numerous criticisms citizens have expressed about the increase in surveillance cameras in the city. Although intended to protect and maintain order on public roads, there have been concerns that images are being misused to monitor private lives and steal private data.

Combined, the presented case studies from different ASEAN cities align with Crumpton’s concerns about the lack of consideration for inter-contextual differences. By imposing the ASCN provisions and focusing on targeted smart city elements, this risks the region falling further from sustainability practices that ensure basic services are provided to people.

## 4. Conclusion

The ‘quintuple helix model’ provided the basis for assessing the nexus between the ASCN and sustainability. Interactions among actors within the state are what establish and strengthen innovation systems, which are essential for generating balanced development approaches. The 2021 study concluded that the vast disparities among Southeast Asian states had severely impeded efforts in sustainable urban planning and governance, affecting the ASCN’s ability to balance development interests with sustainable measures. This study aims to build on past narratives by updating data on the political, social, environmental, and economic landscape of Southeast Asian states between 2020 and 2024, which has severely affected how the region’s ASCN participating cities can incorporate sustainable measures. In terms of the emphasis on economic disparity, additional data on the EoDB among ASEAN member states is also considered.

As predicted, smart city approaches are challenging to adopt in regions with authoritarian or semi-authoritarian settings. Consistent with the conclusions of the previous study, this updated research finds a lack of good governance, accountability, inclusiveness, and transparency among many participating ASCN members. Consequently, no direct correlation is seen between achieving the smart city status and sustainability. As shown by the EIU and WPFI figures presented in this study, democracy and state protection of press freedom remain lacking in many Southeast Asian states. The concern, therefore, is that cities such as Ho Chi Minh City, Hanoi, and Yangon are geared toward achieving smart city status despite lacking the foundational civic and people-to-people relations, raising concerns about the trajectory of their development. The disparity among ASEAN member states is also evident in the global rankings of the EPI and EoDB, with some countries, such as Singapore, ranked among the highest globally. In contrast, others, such as Cambodia, ranked among the lowest in terms of environmental and business landscapes. Unfortunately, the issue found at the state level among ASEAN member states also applies to the participating ASCN cities. The case is clear: Hanoi, Ho Chi Minh City, Phnom Penh, Vientiane, and Myanmar, based on the Globalization and World Cities ranking, have struggled to show greater care for civic rights. Therefore, rather than focusing on addressing such profound neglect of political rights, the focus, as instructed in the ASCN, has been on adopting smart city measures. The fear is that cities will neglect fundamental urban services.

The findings of this study extend the existing discourse on the lack of connection between smart city development programs and sustainability. The term “smart city” is commonly used in contemporary urban planning. However, this severely undermines the long process a city needs to develop. It is as if cities can simply be updated with state-of-the-art technologies. In the case of the ASCN participating cities, the concerns include disparities in capacity, a lack of fundamental civic and human rights, and different problems within the cities. These disparities lead to differences in participation, governance, and socio-ecological sustainable planning, which are not precisely captured by the ASCN’s smart city concept. In the case of ASCN cities in authoritarian and semi-authoritarian settings, there is a tendency for authoritarian systems to deepen amid calls from the central government for greater surveillance and movement control. Amid recent progress in Southeast Asia, a deterioration in sustainability norms is observed, and this trend is likely to continue if the primary narrative driving city-based development is smart city conceptions.

## Declaration of the use of AI

No Generative AI or AI-assisted technologies were used in the writing of this manuscript.

## Data Availability

The data that support the findings of this study can be accessed below:
1. World Economic Outlook 2024 (IMF): https://www.imf.org/en/publications/weo/issues/2024/10/22/world-economic-outlook-october-2024
2. Human Development Index 2022 (UNDP): https://hdr.undp.org/data-center/human-development-index#/indicies/HDI
3. Democracy Index 2024 (Economist Intelligence): https://www.eiu.com/n/campaigns/democracy-index-2024/
4. World Press Freedom 2024 (Reporters Without Borders): https://rsf.org/en/2024-world-press-freedom-index-journalism-under-political-pressure
5. Environmental Performance Index 2024 (Yale University): https://epi.yale.edu/measure/2024/epi
6. Ease of Doing Business 2020 (World Bank): https://archive.doingbusiness.org/en/rankings
7. World Cities 2024 (Globalization and World Cities): https://gawc.lboro.ac.uk/gawc-worlds/the-world-according-to-gawc/world-cities-2024/
