# Supplementary material for: Are Smart Cities ‘Sustainable?’ Revisiting the ‘Helix Model’ of the ASEAN Smart Cities Network
Source: F1000Res. 2026 Sep 8;14:1484. Originally published 2025 Dec 31. [Version 3] doi: 10.12688/f1000research.174616.3 (PMC13570117; doi:10.12688/f1000research.174616.3)
Supplement: Peer review history

# Reviewer report: Reviewer response for version 3

**DOI:** 10.5256/f1000research.209583.r519178

**Published:** 2026-09-11

**Authors:** Tomáš Peráček

**Affiliations:** 1 Comenius University Bratislava, Odbojárov, Bratislava, Slovakia

Dear author,

 I have studied the revised manuscript very carefully.

 Based on the changes and modifications made, I agree to indexing the article.

Is the work clearly and accurately presented and does it cite the current literature?

Yes

If applicable, is the statistical analysis and its interpretation appropriate?

Yes

Are all the source data underlying the results available to ensure full reproducibility?

Yes

Is the study design appropriate and is the work technically sound?

Yes

Are the conclusions drawn adequately supported by the results?

Yes

Are sufficient details of methods and analysis provided to allow replication by others?

Yes

Reviewer Expertise:

smart cities, law, management, sustainability, wehicles, enviroment

I confirm that I have read this submission and believe that I have an appropriate level of expertise to confirm that it is of an acceptable scientific standard.

# Reviewer report: Reviewer response for version 2

**DOI:** 10.5256/f1000research.197926.r493758

**Published:** 2026-07-06

**Authors:** Tomáš Peráček

**Affiliations:** 1 Comenius University Bratislava, Odbojárov, Bratislava, Slovakia

Dear authors,

 Your manuscript is really extremely interesting, up-to-date and very engaging to read. In this case, I recommend making only a few relatively simple adjustments and additions so that the final result of your efforts is perfect.

 Sort keywords appropriately alphabetically.

 The systematics of the manuscript or the labeling of individual chapters does not deviate from the usual practice, but there are minor deviations in the titles of individual chapters. However, this is not a problem. This also indicates that you are following the instructions for authors mentioned on the journal's website. This makes your scientific study homogeneous and forms a well-readable whole.

 The introduction correctly and briefly places the study in the issue and also emphasizes its importance. Despite the stated goal, it would be appropriate to establish hypotheses or research questions. The clarity of the scientific study would also be strengthened by a description of the systematics of your scientific study,

 The theoretical basis is correctly and indeed very necessary as a separate chapter. Nevertheless, I suggest supplementing it with the use of knowledge from other very current and freely available works, e.g. by European authors from the WoS and Scopus databases.

 The methodological chapter would be appropriate to expand with a description of basic scientific research methods such as analysis, synthesis, induction, deduction as done by the authors Peráček & Kaššaj 2026.

 In conclusion, you could answer the established hypotheses/research questions. At the same time, supplement the conclusion with the limits of the study as well as the subject of future research.

Is the work clearly and accurately presented and does it cite the current literature?

Yes

If applicable, is the statistical analysis and its interpretation appropriate?

Yes

Are all the source data underlying the results available to ensure full reproducibility?

Yes

Is the study design appropriate and is the work technically sound?

Yes

Are the conclusions drawn adequately supported by the results?

Yes

Are sufficient details of methods and analysis provided to allow replication by others?

Yes

Reviewer Expertise:

NA

I confirm that I have read this submission and believe that I have an appropriate level of expertise to confirm that it is of an acceptable scientific standard, however I have significant reservations, as outlined above.

## References

[1] : Strategic Management of Urban Services Using Artificial Intelligence in the Development of Sustainable Smart Cities—Managerial and Legal Challenges. *Sustainability* .2026;18(2) : 10.3390/su18020582 10.3390/su18020582

[2] : Transforming European Security: Industrial Resilience, Institutional Adaptation, and Strategic Autonomy for Sustainable Development. *World* .2026;7(5) : 10.3390/world7050070 10.3390/world7050070

# Response

**Published:** 2026-08-28

**Authors:** bama andika putra

**Affiliations:** University of Bristol School of Sociology Politics and International Studies, Bristol, England, UK

Comment 1: Sort keywords appropriately alphabetically.

 Response: Corrected

 Comment 2: The introduction correctly and briefly places the study in the issue and also emphasizes its importance. Despite the stated goal, it would be appropriate to establish hypotheses or research questions. The clarity of the scientific study would also be strengthened by a description of the systematics of your scientific study,

 Response: We agree with this comment, and have decided to revise the following paragraphs. First is the addition of an explicit research question to add clarity to the study’s trajectory. The revisions is located in the last paragraph of the study:

 The intention of this study is thus similar to that of past studies, which aim to reveal the profoundly diverse region of Southeast Asia and the challenges it poses to implementing sustainability measures within the ASCN’s conception of smart cities. Two central questions guide this study. First, to what extent do disparities among the ASEAN member states between 2020 and 2024 affect the capacity to generate sustainability outcomes? Second, what would be the manifestation of that disparity if situated within the context of the ASCN smart city projects?

 Second, at the end of the last paragraph of the introduction section, we have also added additional explantaions related to the roadmap of the article: The following sections first explore the novelty of this study, considering literature on ASEAN, ASCN, smart cities, and sustainability. Meanwhile, the discussions of this study focus on providing the ‘helix model’ analysis, which outlines the recent developments of the ASCN between 2020 and 2024, which goes along with an assessment of the state- and city-level indicators that indicate the diversity among ASEAN member states.

 Comment 3:

 The theoretical basis is correctly and indeed very necessary as a separate chapter. Nevertheless, I suggest supplementing it with the use of knowledge from other very current and freely available works, e.g. by European authors from the WoS and Scopus databases.

 Response: We have incorporated other insights that work on the helix model, specifically in two parts of the study:

 First is the introduction section (paragraph 5, page 2): This article seeks to update Charles David Crumpton’s ‘quintuple helix model’ to assess the nexus between the ASCN and sustainability. The approach is grounded in the ‘helix models’ that symbolize the interactions among different sectors. ^17^^ ,^^18^ The interactions considered include actors in the education, entrepreneurial, public, media, and ecological contexts, ^19^^ –^^23^ which collectively can boost regional innovation systems. The Crumpton-led 2021 study concluded that the diversity found in Southeast Asian states, some of which have authoritarian systems, has severely impeded the “socio-ecologically sustainable urban planning and governance” of the ASCN’s smart cities. ^20^ Past literature has considered the unique insights generated from a quintuple helix model interpretation, with it being implemented across different European Cases in the past (Loučanová et al., 2025, 2026; Taratori et al., 2021)

 Second is the literature review section (2.3), paragraph 1, page 6: Studies in the past have raised doubts about whether smart cities are indeed sustainable means of developing cities. As past studies have shown, there is unclear evidence that shows that the smart city approach indeed leads to urban sustainability. ^1^^ ,^^3^^ ,^^5^^ ,^^63^^ ,^^64^ In building up such an assumption, Charles David Crumpton, Supawatanarkorn Wongthanavasau, Peerasit Kamnuansilpa, John Draper, and Eva Bialobrzeski specifically analyzed this in the context of the ASCN. Adopting the ‘helix models,’ the study looks into specific city plans in Southeast Asia, the ASCN, and experiences with the smart city initiative. ^20^ As also argued in past inquiries (Almeida & Deutsch, 2025; Almulhim & Yigitcanlar, 2025), this study also highlights the disparities in social, economic, and political rights among Southeast Asian states and argues that the presence of states with authoritarian settings generates a more significant challenge for the ASCN. It is then proposed that the smart city conceptualization be within a ‘system of systems’ framework. ^20^ In this, “the complex socio-ecological interaction between society and nature and the multi-layered character of governance are acknowledged”. ^20^

 Comment 4: The methodological chapter would be appropriate to expand with a description of basic scientific research methods such as analysis, synthesis, induction, deduction as done by the authors Peráček & Kaššaj 2026

 Response: We agree with this suggestion, and have added additional explanations regarding the methods that are aligned to Peracek’s 2026 study (paragraph 2, page 3): Through qualitative document analysis, this study aims to update the ASCN under the ‘helix models.’ It considers several unique trends between 2020 and 2024 that contribute to the diversity of Southeast Asian states. These include the Myanmar military coup in 2021, Laos’ foreign debt surges, and Cambodia’s change of leadership after more than three decades, to name a few. ^27^^ –^^33^ Specifically, this study adopts recent methodological practice in the social sciences, such as that introduced in Peráček’s 2026 study (Peráček et al., 2026), which integrates interpretive and comparative analysis in line with literature synthesis by drawing upon the complementary methods of analysis, synthesis, induction, and deduction.

 Comment 5: In conclusion, you could answer the established hypotheses/research questions. At the same time, supplement the conclusion with the limits of the study as well as the subject of future research

 Response: the last two paragraphs of this study covers several of the concerns mentioned with reference to the article’s conclusions:

 This study yields several key insights. First, the political, environmental, economic, and social disparities among ASEAN member states have significantly constrained the ASCN’s capacity to deliver sustainability outcomes. Consequently, as a second insight generated, these disparities have led specific ASCN projects, such as those in Phnom Penh, Hanoi, and Yangon, to risk weak national-level accountability measures and lack of oversight on governance risks.

 This study's findings extend the existing discourse on the disconnect between smart city development programs and sustainability. The term “smart city” is commonly used in contemporary urban planning. However, this severely undermines the long process cities need to develop. It suggests that cities can simply be updated with state-of-the-art technologies. In the ASCN participating cities, concerns include disparities in capacity, a lack of fundamental civic and human rights, and different problems within the cities. These disparities lead to differences in participation, governance, and socio-ecological sustainable planning that the ASCN’s smart city concept does not fully capture. In ASCN cities in authoritarian and semi-authoritarian settings, authoritarian systems tend to deepen amid central-government calls for greater surveillance and control of movement. Amid recent progress in Southeast Asia, there is then a true concern over the deterioration in sustainability norms in accordance to Crumpton’s 2021 framework, despite the task of tracing the deterioration remaining an inquiry for future studies.

# Reviewer report: Reviewer response for version 2

**DOI:** 10.5256/f1000research.197926.r478728

**Published:** 2026-06-02

**Authors:** Eva Kassens-Noor

**Affiliations:** 1 Technical University of Darmstadt, Darmstadt, Darmstadt, Germany

The manuscript has improved in terms of structure and contextual clarity, and the author has addressed most of our comments. However, the central analytical concern remains only partially addressed. While the paper presents relevant descriptive evidence on disparities among ASEAN member states, it still requires a stronger analytical lens to connect these disparities to ASCN smart city implementation and sustainability outcomes.

 For example, the author writes: *“Although the presented data pertain to state actors, there is a strong likelihood that the patterns… are also of concern to local and regional governments.”* With such statements, the author needs to provide a stronger analytical bridge. More specifically, the manuscript should explain more clearly how national-level problems, such as weak democracy, limited press freedom, or poor environmental performance, affect specific ASCN smart city projects in specific cities.

 A similar issue appears in the following paragraph: *“The disparities will only lead to greater differences among ASCN members, given the absence of sustainability measures in ASCN projects. Perhaps two case studies of concern are in Yangon and Phnom Penh. In Yangon, prosecutions continue amid the military junta’s crackdown on democratic voices. Meanwhile, despite the acceleration of development in Phnom Penh, this has been met with an increase in money laundering, concerns over foreign debt, and rising unemployment, indicating that smart cities’ focus on transportation services remains unbalanced with other essential services.”*

 The author’s point is reasonable and relevant, but this paragraph would benefit from a clearer analytical link between the examples provided and the broader argument about ASCN, sustainability, and smart city development. In particular, the discussion should explain more explicitly how the cases of Yangon and Phnom Penh illustrate the absence of sustainability measures in ASCN projects, and how these examples demonstrate the risk of widening disparities among ASCN members.

 In addition, claims such as *“no direct correlation is seen between achieving the smart city status and sustainability,” “the fear is that cities will neglect fundamental urban services,” *and *“a deterioration in sustainability norms is observed”* require stronger empirical support. The paper does not clearly define or test the relationship between “smart city status” and “sustainability,” nor does it provide sufficient evidence to demonstrate a deterioration in sustainability norms over time. These claims should therefore either be supported with clearer empirical evidence or reframed more cautiously as concerns or risks rather than established findings.

Is the work clearly and accurately presented and does it cite the current literature?

Yes

If applicable, is the statistical analysis and its interpretation appropriate?

Not applicable

Are all the source data underlying the results available to ensure full reproducibility?

Yes

Is the study design appropriate and is the work technically sound?

Partly

Are the conclusions drawn adequately supported by the results?

Partly

Are sufficient details of methods and analysis provided to allow replication by others?

Partly

Reviewer Expertise:

Transport and traffic engineering

I confirm that I have read this submission and believe that I have an appropriate level of expertise to confirm that it is of an acceptable scientific standard, however I have significant reservations, as outlined above.

# Response

**Published:** 2026-08-28

**Authors:** bama andika putra

**Affiliations:** University of Bristol School of Sociology Politics and International Studies, Bristol, England, UK

Comment 1: However, the central analytical concern remains only partially addressed. While the paper presents relevant descriptive evidence on disparities among ASEAN member states, it still requires a stronger analytical lens to connect these disparities to ASCN smart city implementation and sustainability outcomes. For example, the author writes: *“Although the presented data pertain to state actors, there is a strong likelihood that the patterns… are also of concern to local and regional governments.”* With such statements, the author needs to provide a stronger analytical bridge. More specifically, the manuscript should explain more clearly how national-level problems, such as weak democracy, limited press freedom, or poor environmental performance, affect specific ASCN smart city projects in specific cities.

 Response: I agree that there needs to be a stronger analytical bridge that allows for clarity relating to the assertions made, and the actual arguments presented. The first revision in page 7 (paragraph 5): Table 1 below shows how diverse Southeast Asian countries are, with greater disparity in recent years. Although the data presented pertain to state actors, there is a strong likelihood that patterns of democracy, GDP growth, human development, press freedom, ease of doing business, and environmental performance at the state level also concern local and regional governments. ASCN projects face similar constraints as those encountered at the state level, where local government bodies are influenced by legal and political challenges. Furthermore, the level of press freedom within a state also says much about the context of local project implementation, including land acquisitions, data use, and procurement.

 Second, I respond to the issue of the connection between how national-level problems, such as weak democracy, limited press freedom, and poor environmental performances, affect specific ASCN city projects. I have dones so within the discussion, but seems to be disconnected due to the separation between Tables 1 and 2. Therefore, I added an additional paragraph clarifiying the association/connection, which is located as the last paragraph of section 3: The findings also indicate a strong link between national-level problems and specific ASCN smart city projects in specific cities. Hanoi’s low scores within the context of EIU/WPFI correlate with the city’s engagement with the surveillance center project. Concerns over Phnom Penh’s low EoDB and HDI scores can also be associated with the city’s decision to establish the ‘Smart City Strategic Planning’ and ‘Build4People’ projects. Like Hanoi, Vientiane’s low EIU score has also led it to pursue projects focused on smart environmental development and sustainable urban transport.

 Comment 2: A similar issue appears in the following paragraph: *“The disparities will only lead to greater differences among ASCN members, given the absence of sustainability measures in ASCN projects. Perhaps two case studies of concern are in Yangon and Phnom Penh. In Yangon, prosecutions continue amid the military junta’s crackdown on democratic voices. Meanwhile, despite the acceleration of development in Phnom Penh, this has been met with an increase in money laundering, concerns over foreign debt, and rising unemployment, indicating that smart cities’ focus on transportation services remains unbalanced with other essential services.”*

 Response: I have revised the paragraph that you are concerned with, looking to connect the national-level discourses to the ASCN city-specific projects. The revised paragraph (paragraph 3, page 11): Perhaps two case studies of concern are in Yangon and Phnom Penh. In Yangon, prosecutions continue amid the military junta’s crackdown on democratic voices. The ‘One Map Yangon,’ for example, a geospatial data-mapping initiative, raises concerns because the junta actively prosecutes dissidents and because such a program increases surveillance and targeted risks. Meanwhile, in the case of Phnom Penh, several national-level concerns, such as foreign debt and money laundering issues, have raised red flags relating to the project’s accountability. Unfortunately, none of the programs listed in the ASCN address concerns over governance and transparency, with the Phnom Penh Walkway and Phnom Penh Public Bus Service programs leaning toward transportation-focused programs.

 Comment 3: The author’s point is reasonable and relevant, but this paragraph would benefit from a clearer analytical link between the examples provided and the broader argument about ASCN, sustainability, and smart city development. In particular, the discussion should explain more explicitly how the cases of Yangon and Phnom Penh illustrate the absence of sustainability measures in ASCN projects, and how these examples demonstrate the risk of widening disparities among ASCN members.

 Response: We agree with this comment, and have added an additional paragraph that highlights the main concerns related to the Phnom Penh and Yangon case studies. Both are united by the absence of sustainability measures, and concerns over increasing disparities among the ASCN members. The revisions (paragraph 4, page 11): Two major concerns emerge in the Yangon and Phnom Penh cases. The first relates to the absence of sustainability measures. As the article defines sustainability as processes of urban planning, management, and policy formulation designed to establish sustainability objectives, both case studies still lack essential elements of transparency, responsiveness, and inclusiveness. Second, both case studies also reflect the wide disparities among the ASCN member states. Cities like Singapore and Jakarta operate under much firmer and more consistent institutional oversight, highlighting the larger gap in the capacities of ASCN members seeking to achieve smart city status.

 Comment 4: In addition, claims such as *“no direct correlation is seen between achieving the smart city status and sustainability,” “the fear is that cities will neglect fundamental urban services,” *and *“a deterioration in sustainability norms is observed”* require stronger empirical support. The paper does not clearly define or test the relationship between “smart city status” and “sustainability,” nor does it provide sufficient evidence to demonstrate a deterioration in sustainability norms over time. These claims should therefore either be supported with clearer empirical evidence or reframed more cautiously as concerns or risks rather than established findings.

 Response: The response is to reframe the claims made, especially in the conclusion section. Three of the last paragraphs at the end of the manuscript are revised:

 As predicted, smart city approaches are challenging to adopt in regions with authoritarian or semi-authoritarian settings. Consistent with the previous study's conclusions, this updated research finds a lack of good governance, accountability, inclusiveness, and transparency among many participating ASCN members. Consequently, the cases and indicators presented in this study do not provide clear evidence that smart city status aligns with outcomes that adhere to sustainability. As the EIU and WPFI figures in this study show, democracy and state protection of press freedom remain lacking in many Southeast Asian states. The concern, therefore, is that cities such as Ho Chi Minh City, Hanoi, and Yangon are geared toward achieving smart city status despite lacking the foundational civic and people-to-people relations, raising concerns about the trajectory of their development.

 The disparity among ASEAN member states is also evident in the global rankings of the EPI and EoDB, with some countries, such as Singapore, ranked among the highest globally. In contrast, others, such as Cambodia, rank among the lowest in environmental and business landscapes. Unfortunately, the issue found at the state level among ASEAN member states also applies to the participating ASCN cities. The case is clear: Hanoi, Ho Chi Minh City, Phnom Penh, Vientiane, and Myanmar, based on the Globalization and World Cities ranking, have struggled to show greater care for civic rights. Therefore, rather than addressing this profound neglect of political rights, the focus, as instructed in the ASCN, has been on adopting smart city measures. The fear is that ASCN member cities such as Ho Chi Minh City, Hanoi, and Yangon risk having fundamental urban services neglected under the label of smart city branding.

 This study's findings extend the existing discourse on the lack of connection between smart city development programs and sustainability. The term “smart city” is commonly used in contemporary urban planning. However, this severely undermines the long process cities need to develop. It suggests that cities can simply be updated with state-of-the-art technologies. In the ASCN participating cities, concerns include disparities in capacity, a lack of fundamental civic and human rights, and different problems within the cities. These disparities lead to differences in participation, governance, and socio-ecological sustainable planning that the ASCN’s smart city concept does not fully capture. In ASCN cities in authoritarian and semi-authoritarian settings, authoritarian systems tend to deepen amid central-government calls for greater surveillance and movement control. Amid recent progress in Southeast Asia, there is then a true concern over the deterioration in sustainability norms in accordance to Crumpton’s 2021 framework, despite the task of tracing the deterioration remaining an inquiry for future studies.

# Reviewer report: Reviewer response for version 2

**DOI:** 10.5256/f1000research.197926.r479775

**Published:** 2026-05-12

**Authors:** Christopher D F Rogers, Marianna Cavada

**Affiliations:** 1 University of Birmingham, Birmingham, UK; 2 Architecture, Manchester School of Architecture, Manchester, England, UK

We find several major problems with this paper.

 The authors state: “smart cities consist of a high quality of life, a competitive economy, and a sustainable environment”. Why are cities that exhibit these qualities smart, and not simply advancing in terms of their sustainability? What is the definition of ‘smartness’ beyond ‘sustainability’? This high-level perspective is important before we get to the specifics and metrics.

 The ASEAN Smart Cities Network (ASCN) is introduced, but the authors have failed to articulate thoroughly what its purpose is (beyond sharing thinking), or what metrics define its success. A more thorough exploration of a common basis for assessment and evaluation of smart initiatives would improve understanding. The same applies to the helix model and its relationship to ASEAN goals. Diagrams may help here in synthesizing the ideas and goals.

 We don’t disagree with any of the quotations from the work on the Helix Model, but it is the attempt to apply it to the ASEAN context that is problematic. Indeed, the authors state: “The basis of the ASCN’s problem is diversity across systems. The smart city approach becomes complex when applied to governments with authoritarian or semi-authoritarian settings, which could lead to further control over populations”.

 Sustainability is determined locally – local conditions set local priorities – and yet here the authors present an amalgam of ten highly variable sets of local conditions and attempts to draw learning from across them. This is in itself highly problematic. Put another way, context is all important and is needed – for example, geographical context to understand the complexities and influences of location in relation to any one smart initiative. This information exists in fragments, but it's scattered and not systematic enough for a wider audience to understand. This extends to their governance systems - an essential aspect of the context.

 More generally, the methodology is not sufficiently clear to provide a robust comparison between smartness and sustainability. Overall, the paper needs a rigorous structure; as it is the paper approaches its task in too general a manner (perhaps because of the very severe challenges it faces) and does not explicitly explain how smartness contributes to the journey towards sustainability (and sustainability is a journey, not an absolute state)  metrics for smartness, and why smartness is of value when the goal is sustainability (as discerned from the arguments advocated in the paper), should be crystal clear.

Is the work clearly and accurately presented and does it cite the current literature?

Partly

If applicable, is the statistical analysis and its interpretation appropriate?

Not applicable

Are all the source data underlying the results available to ensure full reproducibility?

Partly

Is the study design appropriate and is the work technically sound?

No

Are the conclusions drawn adequately supported by the results?

Partly

Are sufficient details of methods and analysis provided to allow replication by others?

No

Reviewer Expertise:

Sustainability, Liveability and Resilience of Cities

We confirm that we have read this submission and believe that we have an appropriate level of expertise to state that we do not consider it to be of an acceptable scientific standard, for reasons outlined above.

# Reviewer report: Reviewer response for version 1

**DOI:** 10.5256/f1000research.192534.r458129

**Published:** 2026-03-12

**Authors:** Eva Kassens-Noor, Hiba Karam

**Affiliations:** 1 Technical University of Darmstadt, Darmstadt, Darmstadt, Germany

The paper argues that smart city development under the ASEAN Smart Cities Network does not necessarily produce sustainability. Instead, regional inequalities and differences in political and governance systems often limit its ability to deliver inclusive and sustainable urban outcomes.

1. Is the work clearly and accurately presented, and does it cite the current literature? partly

Overall, the paper cites current literature, including several recent sources published after 2020, with some references from 2023–2025. This indicates that the manuscript is adequately engaged with recent scholarship. However, these points should be addressed by the author:

- **Abstract:** The abstract is too vague. The abstract identifies a clear research problem; it situates the study in a specific regional context (ASEAN) and signals a theoretical contribution by updating Crumpton’s 2021 quintuple helix model. However, the abstract did not state the main result explicitly. Moreover, “Utilizing qualitative and quantitative methods” is generic; the abstract should briefly indicate what kinds of data or analysis were used.

 

- **Introduction:** The framing would benefit from clearer structure and stronger connections between points. The manuscript currently assumes too much prior knowledge from the reader, particularly regarding the ASEAN Smart Cities Network (ASCN), Crumpton’s 2021 study, and the helix model. More clarity is needed on what ASCN is, what the earlier helix model argued, and why this framework is important for the present study. These concepts should be briefly introduced and linked more clearly to the paper’s overall argument.

 

- **Literature Review: **This literature review is more clearly structured than the earlier framing section, and the subsection organization helps guide the reader through the discussion. However, the overall argument still requires stronger logical connection between points. The section moves across ASEAN norms, sustainability debates, urbanization, ASCN, smart city critiques, and the helix-model framework without always making clear how these elements build on one another. In addition, the manuscript continues to assume prior familiarity with key concepts and prior studies. More clarity is needed on what the ASEAN Smart Cities Network (ASCN) is, how it functions, what the earlier helix-model approach argued, and why that framework is important for the present study. Similarly, Crumpton’s 2021 study should be introduced more fully so that readers can understand its main findings and how the current paper extends it. Overall, the section is relevant and well referenced, but it would benefit from clearer definitions, stronger transitions, and a more precise articulation of the study’s novelty.

 

- **The ‘Helix Model’ of the ASCN: New Observations, Old Patterns: **First, the section still relies too heavily on prior knowledge. Even though subsection 3.2 is called “Revisiting the ‘Helix Model’,” the explanation remains too brief. The reader still needs a clearer account of what the earlier model actually examined, what its dimensions were, what it found, and why it is being reused here. Second, the manuscript repeatedly returns to the same general point: Southeast Asia is diverse, authoritarian settings are problematic, and ASCN may not align with sustainability. Third, phrases such as “what is puzzling,” “profound neglect,” and “may not be the best development course” sound more general and argumentative than rigorously evidenced.

Fourth, the interpretation of Table 1 is not yet fully convincing. While the table does demonstrate substantial disparities across ASEAN member states in terms of human development, democracy, press freedom, environmental performance, and ease of doing business, the discussion tends to move too quickly from descriptive rankings to broader causal conclusions. For example, low scores in the Democracy Index and World Press Freedom Index are used to suggest that ASCN-related smart city development may accelerate surveillance and state control, but this relationship is asserted rather than empirically demonstrated in the analysis presented here. Similarly, differences in EPI and Ease of Doing Business rankings show variation across national contexts, but the link between these indicators and the sustainability or governance outcomes of specific smart city projects requires clearer justification.

 Overall, the section would benefit from a more cautious interpretation of the table, a clearer explanation of why these particular indicators was selected, and a stronger analytical bridge between national-level disparities and claims about city-level smart development.

 The conclusion addresses an important issue and clearly restates the paper’s critical position. However, it is somewhat repetitive and at times more assertive than the analysis supports. In particular, claims about authoritarian deepening, surveillance, neglect of civic rights, and declining sustainability norms should be framed more cautiously unless supported by stronger evidence. The conclusion would be stronger with a more precise synthesis of the main findings, a clearer statement of the paper’s contribution, and a better distinction between findings, interpretation, and broader concerns.

Is the work clearly and accurately presented and does it cite the current literature?

Yes

If applicable, is the statistical analysis and its interpretation appropriate?

Not applicable

Are all the source data underlying the results available to ensure full reproducibility?

Yes

Is the study design appropriate and is the work technically sound?

Partly

Are the conclusions drawn adequately supported by the results?

Partly

Are sufficient details of methods and analysis provided to allow replication by others?

Partly

Reviewer Expertise:

Transport and traffic engineering

We confirm that we have read this submission and believe that we have an appropriate level of expertise to state that we do not consider it to be of an acceptable scientific standard, for reasons outlined above.

# Response

**Published:** 2026-03-19

**Authors:** bama andika putra

**Affiliations:** University of Bristol School of Sociology Politics and International Studies, Bristol, England, UK

**Comment 1:**

**Abstract:** The abstract is too vague. The abstract identifies a clear research problem; it situates the study in a specific regional context (ASEAN) and signals a theoretical contribution by updating Crumpton’s 2021 quintuple helix model. However, the abstract did not state the main result explicitly. Moreover, “Utilizing qualitative and quantitative methods” is generic; the *abstract should briefly indicate what kinds of data or analysis were used.*

**Response**: I have revised the abstract section to explain that the article is a qualitative document analysis, which utilizes secondary data published on the ten ASEAN member states (between 2020 and 2024). This should serve as an update of Crumpton’s 2021 study, which mainly asserts that there are significant diversities found among ASEAN member states, and this contributes towards the diverse social, political, economics and environmental dimensions of the city’s developments (later on argued as affecting the local actor’s capacity in introducing sustainable measures, as smart city conception’s have limited capacity to enforce these measures). The revised abstract (page 1):

 Recent urban planning in Southeast Asia has incorporated the theme of transforming cities to ‘smart city’ status. However, the central question raised in this study is whether the ASEAN’s conception of smart cities is genuinely sustainable. Based on the backdrop of Southeast Asia’s disparity in economic performances and diversity in its political, social, economic, and environmental dimensions, this study updates Crumpton’s 2021 study on the ‘quintuple helix model’ and argues that new observations but old patterns are found in the ASEAN Smart Cities Network’s way of developing cities in Southeast Asia. Through a qualitative document analysis, utilizing published secondary data on the ten ASEAN member states between 2020 and 2024, this study revisits and updates the current state of Southeast Asia and how this corresponds to the challenges of constructing smart cities in democratic, semi-authoritarian, and authoritarian settings.

**Comment 2:**

 Introduction: The framing would benefit from clearer structure and stronger connections between points. The manuscript currently assumes too much prior knowledge from the reader, particularly regarding the ASEAN Smart Cities Network (ASCN), Crumpton’s 2021 study, and the helix model. More clarity is needed on what ASCN is, what the earlier helix model argued, and why this framework is important for the present study. These concepts should be briefly introduced and linked more clearly to the paper’s overall argument.

 Response 1: The revisions to the introduction section can be traced to the first two pages, where the focus is on clarifying what ASCN and Crumpton’s 2021 study consisted of. On the first point on ASCN, the second, third, and fourth paragraphs of page 2 provide several basic pieces of information for readers as to what ASCN is, and the expectations once a city becomes a pilot project city. It traces the history (since 2018), the cities incorporated into the current status quo, and the ASCN's expectations for catalyzing bankable projects to solve urban problems through technology. The third paragraph, on the other hand, looks at the strategic outcomes of the ASCN, which focus on a high quality of life, a competitive economy, and a sustainable environment. The following are the complete paragraphs explaining this point: Among the regions that have actively adopted this smart city concept is Southeast Asia. Under the Association of Southeast Asian Nations (ASEAN), particularly during Singapore’s ASEAN chairmanship in 2018, member states agreed with a list of 26 pilot projects under the ASEAN Smart Cities Network (ASCN). 6 The growth of cities in ASEAN is unique, as they will “continue to be driven by urban centers, with more people expected to urbanize by 2030 and ‘middleweight’ cities of between 200,000 and 2 million residents forecast to drive 40% of the region’s growth”. 7 The role of ASCN is thus to act as a normative guideline for ASEAN member states on smart city development, sharing best practices, and linking partnerships to accelerate growth. 6, 8 The expectation is that the ASCN will act as a facilitator, linking city government officials and business entities to “catalyze bankable projects to solve urban problems by utilizing technology and innovation opportunities provided by the private sector”. 9 ; For ASEAN, the strategic outcomes of smart cities consist of a high quality of life, a competitive economy, and a sustainable environment. 6 To achieve them, several development focus areas have been determined: civic and social, health and well-being, safety and security, quality environment, built infrastructure, and industry and innovations. The determined development areas are enabled through ASEAN’s encouragement of digital infrastructure and applications, and partnership and funding schemes under the ASCN. 10

 Response 2: I have revised the introduction towards Crumpton’s 2021 study by elaborating what were the main findings of this study. First, it looks at how the diversities found in the ASEAN member states increasingly impede the ASEAN member state’s capacity to balance between technological developments, and the provision of basic urban needs/facilities. In the last paragraph of page 2, the additional narratives highlights how inter-contextual differences leads states to struggle to provide the balanced sustainability measures in dimensions of governance, environment, and human rights. Therefore, the key take away of this is that this study enhances the previous finding to show that the disparities among ASEAN member states have continued to take place between 2020 and 2024, and looking at specific case studies of cities in the ASCN, would show how the disparity of the state’s capacities leads to heightened concerns and doubts that local governments prioritize sustainable measures. The revised paragraph (last paragraph page 2): “In the ASCN’s development focus area of civic and social, for example, some countries, such as Vietnam, Laos, Cambodia, and Brunei Darussalam, still lack the basic features of democracy. 24– 26 Thus, the intention of its cities to transform into smart cities is seen as a potential policy to further impinge upon participatory and socio-ecological sustainable urban planning and governance. 20 Therefore, as seen in the case of the ASCN in recent years, Crumpton highlights the emergence of the state under-prioritizing the environment and the lack of initiatives in improving basic services. This lack of adequate consideration of the inter-contextual differences among ASEAN member states means that members would struggle to achieve this smart city status, due to the underlying issue of unmet urban service requirements.”

**Comment 3:**

 Literature Review: This literature review is more clearly structured than the earlier framing section, and the subsection organization helps guide the reader through the discussion. However, the overall argument still requires stronger logical connection between points. The section moves across ASEAN norms, sustainability debates, urbanization, ASCN, smart city critiques, and the helix-model framework without always making clear how these elements build on one another. In addition, the manuscript continues to assume prior familiarity with key concepts and prior studies. More clarity is needed on what the ASEAN Smart Cities Network (ASCN) is, how it functions, what the earlier helix-model approach argued, and why that framework is important for the present study. Similarly, Crumpton’s 2021 study should be introduced more fully so that readers can understand its main findings and how the current paper extends it. Overall, the section is relevant and well referenced, but it would benefit from clearer definitions, stronger transitions, and a more precise articulation of the study’s novelty.

 Response: I make the revisions in the first paragraph of section 2, and the last paragraph of the same section. In the first paragraph (page 3), the focus of the revisions is to highlight why these three introduced themes in the literature review is important for this study, and in what way does the article contribute towards these vast discussions on matters of smart cities and sustainability in the context of Southeast Asia. Meanwhile, the last paragraph of section 2 (page 6) emphasizes the novelty offered, which includes the covering of more empirical case studies that occurred between 2020 and 2024, to showcase whether Crumpton’s assumpions in 2021 is still valid in Southeast Asia.

 1.         Revision 1 (paragraph 1 of page 3): Against the backdrop of unique norms and differences among states, adopting sustainability measures in Southeast Asia poses challenges. The existing literature highlights these challenges well, focusing on the trajectory of sustainability among ASEAN member states over the past years. Nevertheless, not enough attention has been paid to ASEAN’s development pathway, especially given its recent emphasis on advancing it through the construction of smart cities across the region. This section provides a brief literature review of how Southeast Asia engages with sustainability demands. First, it examines the challenges of adopting sustainability across different regional sectors and the tendency in the literature to call for modified governance and policies to address these concerns. Second, this section examines how the ASCN has been one of ASEAN’s solutions to address urbanization issues in Southeast Asia’s larger cities, focusing on smart city initiatives as the basis for these solutions. Lastly, it will revisit recent studies on smart cities and sustainability in Southeast Asia and the novelty this study offers. These three discourses allow for a more nuanced understanding of the dominant discussions on smart cities and sustainability in Southeast Asia, and of the importance of revisiting more recent empirical case studies to strengthen or challenge past interpretations.

 2.         Revision 2 (last paragraph of section 2, page 6): This study builds on the previous concerns on the nexus between smart cities and sustainability in Southeast Asia. Between 2020 and 2024, many developments have occurred that have contributed to capacity disparities, political tensions, and the infringement on social and human rights. Unfortunately, to balance development and sustainability, ASEAN has focused on the ASCN as one of the primary means of achieving balanced development for the region’s larger cities. The novelty of this study thus offers the opportunity to reveal the profound problems associated with the ASCN, which distances itself from actual sustainability measures as its primary aim and further impacts participatory and socio-ecological sustainable urban planning and governance. In doing so, it follows the 2019 framework and highlights how the region’s socio-political developments have contributed to the disparities.

**Comment 4:**

 The ‘Helix Model’ of the ASCN: New Observations, Old Patterns: First, the section still relies too heavily on prior knowledge. Even though subsection 3.2 is called “Revisiting the ‘Helix Model’,” the explanation remains too brief. The reader still needs a clearer account of what the earlier model actually examined, what its dimensions were, what it found, and why it is being reused here. Second, the manuscript repeatedly returns to the same general point: Southeast Asia is diverse, authoritarian settings are problematic, and ASCN may not align with sustainability. Third, phrases such as “what is puzzling,” “profound neglect,” and “may not be the best development course” sound more general and argumentative than rigorously evidenced.

 Response: Revisions can be seen in paragraphs 2 and 3 in the section ‘3.2. Revisiting the ‘Helix Model’’. In the first paragraph, I clarify that one of the elements of Crumpton’s 2021 study is that the Helix Model adopted results based on the assumption of diversity across political, social, and economic dimensions among ASEAN member states. As a consequence, there are different capacities to comply with the ASCN provisions, and a deep concern that, because half of ASEAN members are non-democratic nations, the idea of establishing smart cities would exacerbate human rights conditions in those cities. The argument, therefore, is that the tables presented show this concern over human rights and politics, and argue why there is a lack of care towards sustainability measures in those particular cities. The revised paragraph: “The basis of the ASCN's problem is diversity across systems. The smart city approach becomes complex when applied to governments with authoritarian or semi-authoritarian settings, which could lead to further control over populations. 76 For the more democratic nations of Southeast Asia, the problem then becomes to what extent state policymakers consider environmental costs as exceeding development targets. This section argues that old patterns are emerging, with a lack of relationship between citizens and the smart city developments taking place in Southeast Asia. In doing so, the following will explain several indicators that show the diversity between states and cities in Southeast Asia, indicating different starting points among the ASCN members, which risks exacerbating disparities in the delivery of basic urban services. Similar to Crumpton’s 2021 point, the updated empirical data presented between 2020 and 2024 in the two tables below show that concern over non-democratic practices has the potential to negatively affect urban planning and governance, underplay environmental concerns, and neglect improvements in the quality of life of the city’s population. ”

**Comment 5: **the interpretation of Table 1 is not yet fully convincing. While the table does demonstrate substantial disparities across ASEAN member states in terms of human development, democracy, press freedom, environmental performance, and ease of doing business, the discussion tends to move too quickly from descriptive rankings to broader causal conclusions. For example, low scores in the Democracy Index and World Press Freedom Index are used to suggest that ASCN-related smart city development may accelerate surveillance and state control, but this relationship is asserted rather than empirically demonstrated in the analysis presented here. Similarly, differences in EPI and Ease of Doing Business rankings show variation across national contexts, but the link between these indicators and the sustainability or governance outcomes of specific smart city projects requires clearer justification. Overall, the section would benefit from a more cautious interpretation of the table, a clearer explanation of why these particular indicators was selected, and a stronger analytical bridge between national-level disparities and claims about city-level smart development.

 1.         Response 1: I make the connection between variation across national contexts, with the issue of sustainability in the smart city projects. In doing this, explanations in pages 10 and 11 emphasizes how in several case studies, the focus on smart city status (mainly transportation and technological developments within the cities) have neglected the basic services and human rights issues that the cities and state actors are suppose to focus on in the first place. The revised paragraphs, and the insertion of case studies in section 3:

 (paragraph 1 page 10): However, what is puzzling is the rising significance of cities in less democratic settings such as Hanoi, Ho Chi Minh City, Phnom Penh, Vientiane, and Myanmar. In the past four years, these four cities have struggled to ensure greater civic rights are maintained. As the ASCN is focused on accelerating the efforts to make these cities ‘smart,’ there seems to be a profound neglect of the political issues in those cities due to this dominant discourse of ‘smart’ development. This raises a substantial concern, as these cities would focus solely on consistency with the ASCN smart city framework, thereby further neglecting fundamental urban services. In Vientiane and large cities in Laos, the concern still lies on the issue of restricted civil and political rights, which includes cases of unfair criminal trials, and judicial corruption in the cities (HRW, 2017; OHCHR, 2024). Meanwhile, in Jakarta, opponents of democratic rule have been met with harsh measures to suppress negative sentiment toward the president (Raditio and Yeremia, 2022; BBC, 2026). These cases go to show that accelerating development that leans to being ‘smart’ does not automatically translate into the provision of basic political and human rights services being delivered. It also shows a strong linkage between state-level political concerns and local-level appreciation towards human rights.

 (paragraph 1, page 11): The ASCN does not consider these capacity disparities. The focus has been on accelerating the ‘smart city’ development of the ASCN’s pilot city projects and on leveraging AI and technology to deliver better public services to the city’s population. However, the complexity is that cities in countries without democratic systems show less appreciation for the environment and lack proper prospects for human development. The updated data from the 2020 study show that the same conclusions have been reached. The disparities will only lead to greater differences among ASCN members, given the absence of sustainability measures in ASCN projects. Perhaps two case studies of concern is in Yangon and Phnom Penh. In Yangon, prosecutions continue amid the military junta’s crackdown on democratic voices (Oponio Juris, 2022; Reuters, 2022). Meanwhile, despite the acceleration of development in Phnom Penh, this has been met with an increase in money laundering, concerns over foreign debt, and rising unemployment, indicating that smart cities’ focus on transportation services remains unbalanced with other essential services.

 (paragraph 3, page 11): The problem with diverse landscapes such as ASEAN is that ‘smart’ solutions can easily deepen authoritarian tendencies and undermine sustainability objectives. 20 Another issue is the lack of acknowledgment that the starting points of these cities are different, with some already building the foundations of smart cities since the 1960s. 90 As a consequence, it is expected that, after observing recent data in the past four years, poor cities would most likely see a more substantial deterioration of sustainability norms, as anticipated in past studies. 92– 94 The case of Hanoi, Vietnam, is interesting, given the numerous criticisms citizens have expressed about the increased number of surveillance cameras in the city. Although intended to protect and maintain order on public roads, there have been concerns that images are being misused to monitor private lives and steal private data (Dan, 2026).

 (paragraph 4, page 11): Combined together, the presented case studies from different ASEAN cities shows an alignment with Crumpton’s concerns over the lack of consideration towards inter-contextual differences. By imposing the ASCN provisions and focusing on targeted smart city elements, this risks the region falling further from sustainability practices that ensure basic services are provided to people.

 2. Response 2: On the point of national-level disparities and claims about city-level smart development, I have argued in paragraph 3 of section 3.2, that because of there is a strong linkage with the social issues faced in the national level, to the social realities in local and regional levels (especially in non-democratic states). The revised paragraph: “Table 1 below shows how diverse Southeast Asian countries are, showing more significant disparity in the past years. Although the presented data pertain to state actors, there is a strong likelihood that the patterns of democracy, GDP growth, human development, press freedom, ease of doing business, and environmental performance at the state level are also of concern to local and regional governments. This considers that half of Southeast Asian states are non-democratic, suggesting that a hierarchical system will apply to the concerns that government officials focus on.  The indicators consider several dimensions pivotal to the construction of smart cities. First is the 2024 GDP growth, published by the International Monetary Fund, and considers the total value of goods and services produced within the country. 77 Second is the 2022 UN Human Development Index (HDI), which considers education, health, and standard of living. 78 Third is the 2024 Economist Intelligence Unit’s Democracy Index (EIU), which considers civil liberties, the electoral process, political participation & culture, and the functioning of the government. 79 Fourth is the 2024 Reporters Without Borders’ World Press Freedom Index (WPFI), which ranks states based on assessments by non-governmental organizations of journalists' and media freedom. 80 Fifth is the 2024 Yale University’s Environmental Performance Index (EPI), which considers the state of sustainability (climate change performance, environmental health, ecosystem vitality). 81 Last, which differs from the datasets used in the 2021 study helix model assessment on ASCN, is the 2020 World Bank’s EoDB ranks, which rate the difficulty of starting a business, obtaining construction permits, accessing electricity, registering property, and obtaining credit. 82 ”

# Reviewer report: Reviewer response for version 1

**DOI:** 10.5256/f1000research.192534.r451036

**Published:** 2026-02-02

**Authors:** Yuni Intarti

**Affiliations:** 1 Universitas Indonesia, Depok, West Java, Indonesia

**Is the work clearly and accurately presented and does it cite the current literature?**

**Partly**

 The manuscript’s narrative objective is clearly delineated in both the abstract and introduction: it seeks to "revisit and update" a prior critique of the helix model concerning ASCN sustainability by integrating recent regional developments. This study references a combination of foundational critiques of smart cities and discussions on region-specific governance and human rights, which is appropriate for the subject and indicates an effort to engage with both historical and contemporary scholarship.

 Major numeric and source inconsistencies are listed in Table 1 (critical The paper labels "Ease of Doing Business 2024 (World Bank)" and this reflects the current (2021–2024) context. However, the World Bank’s Doing Business "rankings" page indicates that the rankings are benchmarked to May 2019, with the archive explicitly containing "complete historical data up to Doing Business 2020." This discrepancy is not a minor naming issue: if an indicator is based on 2019/2020, it cannot validly support claims about "the past four years" without strong justification and careful wording. More importantly, at least two entries in Table 1 are demonstrably incorrect when compared to the official Doing Business 2020 ranking table: the manuscript reports an "EoDB global rank" of 187 for Cambodia and 187 for Vietnam. The Doing Business 2020 ranking table lists Cambodia at 144 and Vietnam at 70. This is a direct data error that undermines the subsequent interpretation of "ease of doing business" disparities and any argument built on those values.

**Please check the following source: https://archive.doingbusiness.org/en/rankings?region=east-asia-and-pacific**

 Missing HDI value for Vietnam (critical) The manuscript lists Vietnam’s HDI as "NA." However, UNDP’s own country communication reporting the 2022 HDI states that Vietnam’s 2022 HDI value is 0.726 with a rank of 107/193. This indicates a data extraction, transcription, or table-construction error that should be corrected and documented transparently.

**Check this source: https://www.undp.org/vietnam/press-releases/viet-nam-high-human-development-category-undp-new-report#:~:text=Rich%20countries%20are%20experiencing%20record,of%20193%20countries%20and%20territories.**

 Conceptual clarity gap: "qualitative and quantitative methods" without description. While the manuscript frequently describes itself as using mixed approaches, it does not provide a clear, explicit methods section that explains the qualitative component (e.g., document analysis, case material selection, coding approach) and how it is integrated with the quantitative component. This makes the "presentation" less transparent, even if the prose is readable.

 Adopting methodological and measurement standards is advisable to enhance the positioning of the literature. Given that the primary argument of the manuscript hinges on the sustainability of "smart city" initiatives, incorporating established indicator standards and measurement frameworks specific to urban contexts, rather than those applicable solely to countries, would be advantageous. Examples of such standards include the ISO standards for city services and quality of life, as well as smart cities (ISO 37120:2018 and ISO 37122:2019), and global KPI frameworks for SSCs, such as the ITU-U4SSC KPIs. Although their absence is not necessarily detrimental, it contributes to the uncertainty regarding the operational definition of "sustainability" within the paper.

**Refer to https://cdn.standards.iteh.ai/samples/68498/f4d5a199575f4f5782d4014dad38029e/ISO-37120-2018.pdf.**

**https://cdn.standards.iteh.ai/samples/69050/2e8b58add7054523af3c08edd7ed571b/ISO-37122-2019.pdf**

**https://u4ssc.itu.int/u4ssc-kpi/**

**Is the study design appropriate and is the work technically sound?**

**Partly**

 Using a cross-national, indicator-based comparative methodology is technically appropriate for a scoping or diagnostic paper that aims to map differences across countries and highlight governance or sustainability issues within regional policy frameworks. In this context, comparing governance, development, environmental factors, and press freedom is a reasonable descriptive approach. However, several design choices compromise the technical robustness of the paper’s primary question—“Are smart cities sustainable?”—and the proposed helix-model framework:

 Construct-to-measurement mismatch (critical). The conclusions of this study on sustainability are primarily derived from country-level indices (HDI, democracy, press freedom, EPI, etc.) and lists of ASCN projects. This approach risks ecological inference problems, where relationships observed at the country level may not apply to city-level patterns or project-level sustainability outcomes. This risk of “ecological fallacy” is well-known in aggregate-level inference work and should be explicitly addressed through design, limitations, and ideally, multi-level data.

 Helix-model integrity problem: Excluding the education helix while retaining the “quintuple helix” language (critical). The article mentions excluding the education stakeholder helix due to an “inconclusive relationship” between ICT projects and top-ranked universities. However, foundational descriptions of the Quintuple Helix model consider the education system as one of the five helices; removing it alters the theoretical framework and necessitates re-specification and justification. If the paper employs a modified helix model, it should be renamed (e.g., “modified quadruple helix” or “adapted helix framework”) and explicitly linked to indicators (construct a diagram and a table that associate each helix with its respective indicator(s), data source, and projected relationship to sustainability). If the education helix is limited, the framework is renamed and the theoretical implications are elucidated, referencing the definitions of the Quintuple Helix).

 The unit of analysis is under-specified. The manuscript shifts between discussing: - ASEAN member states (country-level governance, environmental performance, and development), - ASCN cities (GaWC categorization and project lists), and - “smart city sustainability” as an outcome concept. This could be coherent, but only if the design clearly specifies: (a) the primary unit of analysis, (b) the linking logic between country indices and city/project outcomes, and (c) how alternative explanations are addressed.

 The interaction with the ASEAN Smart Cities Network normative framework is limited. The ASEAN Smart Cities Framework equates an ASEAN smart city with a smart sustainable city, emphasizing a balance among economic, environmental, and quality of life considerations while committing to an inclusive approach that respects human rights and fundamental freedoms. A robust critique of ASCN’s sustainability would benefit from a clear contrast between the framework’s stated commitments and evidence of implementation gaps, such as those found in project design features, governance mechanisms, and monitoring indicators. The current manuscript alludes to this gap but does not systematically address it.

**Are sufficient details of the methods and analysis provided to allow replication by others?**

**No**

 Engagement with the normative framework of the ASEAN Smart Cities Network (ASCN) is limited. The ASEAN Smart Cities Framework equates an ASEAN smart city with a sustainable smart city. However, a clear data-processing pathway is absent. The methods by which raw data from each source were extracted (whether through a web interface or downloadable tables), cleaned, standardized across different year baselines, and converted into the reported values (scores and ranks) are not elucidated. The evident errors in Table 1 further underscore the need for a transparent verification pipeline.

 Additionally, no explicit strategy exists for addressing missing data. Values labeled as “NA” (such as Vietnam HDI and Brunei democracy in Table 1) lack accompanying rules regarding whether countries are excluded from certain analyses, whether the absence is due to unavailable metrics, or if it indicates a data-handling error. Furthermore, there is no description of the qualitative method (it is imperative to publish a "Table 1 & Table 2 dataset" file (in CSV/XLSX format) alongside a code script capable of regenerating the tables from raw data whenever feasible). This is particularly significant because the paper focuses on derived tabular results, addressing issues such as the reasons for missing data, its impact, and management strategies, including corrections for instances where "missing" data is actually an error (e.g., Vietnam HDI).

  If the manuscript employs qualitative evidence (as claimed), replicability necessitates at least the following: criteria for corpus selection (which documents/events and why), the analytic procedure (coding, thematic analysis, narrative synthesis), and how qualitative insights were utilized to interpret quantitative patterns. Reporting guidance for qualitative work (SRQR/COREQ, depending on the method) highlights the types of details required by readers to critically evaluate and replicate the analytic logic. (incorporating the study design label, units of analysis, inclusion and exclusion criteria, specific dataset editions or years, extraction dates, variable definitions, and a comprehensive step-by-step guide for constructing tables is essential. Reporting guideline structures, such as STROBE for observational quantitative studies, SRQR/COREQ for qualitative research, and GRAMMS for mixed methods, are employed as practical checklists.

**Is the statistical analysis and its interpretation appropriate, if applicable?**

**No**

 It is imperative to either eliminate the use of correlation terminology and clearly present the findings as descriptive or interpretive or to include a well-defined statistical analysis plan, such as Spearman correlations on ranks with appropriate caveats, discussion of uncertainty, and sensitivity analyses. The chosen approach should be consistent with the evidence presented in the manuscript.

 Provide an operational definition of "smart city status," which is critical. If this status is represented by GaWC categorization, ASCN project counts, project maturity, or another metric, it should be precisely specified and its use justified.

 Incorporate robustness checks to demonstrate how conclusions vary if (a) business environment data are substituted (e.g., Doing Business vs BREADY), (b) ranks versus scores are employed, and (c) alternative sustainability measures are used, such as city-level sustainability KPIs where applicable.

**Are all the underlying source data available to ensure full reproducibility?**

**Partly**

 The paper includes a data availability statement that details the public web sources employed for each index, which is a commendable step toward transparency.

  However, full reproducibility, particularly on an open research platform, is not entirely achieved. The primary issue is that the dataset compiled by the authors, which provides the specific values in Tables 1 and 2, is not stored in a repository with a persistent identifier, and the computational methods used to generate the tables are not disclosed.

 Even when the primary sources are from third parties, the dataset derived by the authors (which is cleaned, harmonized, and compiled from various sources) constitutes a research output that should be shared to facilitate verification, especially given the identified errors in the tables.

 Additionally, some sources may not be readily reusable (for example, accessing the Democracy Index might require submitting a request form), and some indices are updated annually. Therefore, ensuring reproducibility necessitates versioning, recording extraction dates, and archiving specific dataset editions.

**Are the conclusions drawn adequately supported by the results?**

**Partly**

 The descriptive aspect of the argument—“ASEAN is diverse, and governance/environmental baselines differ substantially among member states”—is well aligned with the comparative indices presented in the paper. The claim that authoritarian or semi-authoritarian governance contexts can influence the adoption of “smart” technologies is credible and substantiated by the cited literature.

 However, there is a critical overreach beyond the evidence presented (the assertion that “no direct correlation is seen” between smart city status and sustainability implies an empirical examination of this relationship, yet the manuscript neither conducts such an examination nor defines the necessary variables). Similarly, the claim that a “deterioration of sustainability norms is observed” an empirical trend, but the paper lacks time-series evidence of changing norms.

 There is an insufficient linkage between ASCN projects and sustainability outcomes (although the manuscript lists projects and city classifications (GaWC), it does not evaluate project outcomes against sustainability criteria (e.g., emissions, service equity, resilience, governance safeguards). Without these outcome measures, the conclusion that ASCN “may not be the best development course” remains a reasoned hypothesis rather than a substantiated result.

 It is necessary to address ASCN’s stated principles. The ASEAN Smart Cities Framework explicitly advocates an inclusive approach that respects human rights and defines a “smart city” as a “smart sustainable city.” A conclusion that the ASCN’s concept is not sustainable would be more compelling if it explicitly demonstrated a divergence between these stated principles and observed implementation patterns (e.g., whether “city surveillance” projects include rights protections or whether “quality environment” projects measurably improve environmental outcomes).

Is the work clearly and accurately presented and does it cite the current literature?

Partly

If applicable, is the statistical analysis and its interpretation appropriate?

No

Are all the source data underlying the results available to ensure full reproducibility?

Partly

Is the study design appropriate and is the work technically sound?

Partly

Are the conclusions drawn adequately supported by the results?

Partly

Are sufficient details of methods and analysis provided to allow replication by others?

No

Reviewer Expertise:

border areas (borderland), ASEAN, European Union-ASEAN relations,

I confirm that I have read this submission and believe that I have an appropriate level of expertise to confirm that it is of an acceptable scientific standard, however I have significant reservations, as outlined above.

# Response

**Published:** 2026-03-19

**Authors:** bama andika putra

**Affiliations:** University of Bristol School of Sociology Politics and International Studies, Bristol, England, UK

**Comment 1:**

 Major numeric and source inconsistencies are listed in Table 1 (critical The paper labels "Ease of Doing Business 2024 (World Bank)" and this reflects the current (2021–2024) context. However, the World Bank’s Doing Business "rankings" page indicates that the rankings are benchmarked to May 2019, with the archive explicitly containing "complete historical data up to Doing Business 2020." This discrepancy is not a minor naming issue: if an indicator is based on 2019/2020, it cannot validly support claims about "the past four years" without strong justification and careful wording. More importantly, at least two entries in Table 1 are demonstrably incorrect when compared to the official Doing Business 2020 ranking table: the manuscript reports an "EoDB global rank" of 187 for Cambodia and 187 for Vietnam. The Doing Business 2020 ranking table lists Cambodia at 144 and Vietnam at 70. This is a direct data error that undermines the subsequent interpretation of "ease of doing business" disparities and any argument built on those values.

**Response** : I have corrected the numeric and source inconsistencies listed in Table 1, including revisions with the narratives:

 1.         Rather than stating that the sources are taken between 2021-2024, the beginning of the last paragraph of page 7 explains that the data displays disparity in recent years: “Table 1 below shows how diverse Southeast Asian countries are, showing more significant disparity in the past years.”

 2.         Correction of the EoDB’s year of publication becoming 2020 (last sentence of the last paragraph, page 7): “Last, which differs from the datasets used in the 2021 study helix model assessment on ASCN, is the 2020 World Bank’s EoDB ranks, which rate the difficulty of starting a business, obtaining construction permits, accessing electricity, registering property, and obtaining credit”

 3.         EoDB Global rank data: Cambodia ranked 144, and Vietnam ranked 70

 4.         Narrative in the first paragraph of page 9: “Furthermore, the EoDB figures shown in Table 1 indicate the role of political and government systems in the ease of doing business in ASEAN countries. Singapore is the second-best country in the world to do business, but its ASEAN counterpart, Cambodia, is ranked 144th, among the lowest”

**Comment 2:**

 Missing HDI value for Vietnam (critical) The manuscript lists Vietnam’s HDI as "NA." However, UNDP’s own country communication reporting the 2022 HDI states that Vietnam’s 2022 HDI value is 0.726 with a rank of 107/193. This indicates a data extraction, transcription, or table-construction error that should be corrected and documented transparently.

 Response: I have corrected the data entry for Vietnam’s HDI value in 2022: HDI value 0,726, ranked 107/193. The revisions can be seen in table 1

**Comment 3:**

 Conceptual clarity gap: "qualitative and quantitative methods" without description. While the manuscript frequently describes itself as using mixed approaches, it does not provide a clear, explicit methods section that explains the qualitative component (e.g., document analysis, case material selection, coding approach) and how it is integrated with the quantitative component. This makes the "presentation" less transparent, even if the prose is readable.

 Response: Several sections of the study has been revised to make clear that the qualitative component of the study is through the use of document analysis:

 1.         Abstract: “Recent urban planning in Southeast Asia has incorporated the theme of transforming cities to ‘smart city’ status. However, the central question raised in this study is whether the ASEAN’s conception of smart cities is genuinely sustainable. Based on the backdrop of Southeast Asia’s disparity in economic performances and diversity in its political, social, economic, and environmental dimensions, this study updates Crumpton’s 2021 study on the ‘quintuple helix model’ and argues that new observations but old patterns are found in the ASEAN Smart Cities Network’s way of developing cities in Southeast Asia. Through a qualitative document analysis, utilizing published secondary data on the ten ASEAN member states between 2020 and 2024, this study revisits and updates the current state of Southeast Asia and how this corresponds to the challenges of constructing smart cities in democratic, semi-authoritarian, and authoritarian settings.

 2.         Pages 2-3: “Through quantitative and qualitative approaches (document analysis), this study intends to provide an update on the ASCN under the ‘helix models.’ It considers several unique trends between 2020 and 2024 that contribute to the diversity of Southeast Asian states. These include the Myanmar military coup in 2021, Laos’ foreign debt surges, and Cambodia’s change of leadership after more than three decades, to name a few. 27– 33

**Comment 4:**

 Adopting methodological and measurement standards is advisable to enhance the positioning of the literature. Given that the primary argument of the manuscript hinges on the sustainability of "smart city" initiatives, incorporating established indicator standards and measurement frameworks specific to urban contexts, rather than those applicable solely to countries, would be advantageous. Examples of such standards include the ISO standards for city services and quality of life, as well as smart cities (ISO 37120:2018 and ISO 37122:2019), and global KPI frameworks for SSCs, such as the ITU-U4SSC KPIs. Although their absence is not necessarily detrimental, it contributes to the uncertainty regarding the operational definition of "sustainability" within the paper.

 Response: I do agree that the term sustainability of smart city initiatives deserves closer examination regarding its operationalization. I, however, disagree that the article would benefit from a standardized definition, as proposed (ISO 37120:2018 and ISO 37122:2019). As clearly stated in the following two paragraphs (pages 5-6), the term sustainability in the context of Southeast Asian states is different. The highlight is on the character of governance introduced, as well as heightened concerns about surveillance and control. This follows up on earlier studies on the unique challenges of sustainability in Southeast Asia, and the plan of this study is to build on those findings:

 1.         Paragraph 1: “Studies in the past have captured the doubt that smart cities are indeed sustainable means of developing cities. As past studies have shown, there is unclear evidence that shows that the smart city approach indeed leads to urban sustainability. 1, 3, 5, 63, 64 In building up such an assumption, Charles David Crumpton, Supawatanarkorn Wongthanavasau, Peerasit Kamnuansilpa, John Draper, and Eva Bialobrzeski specifically analyzed this in the context of the ASCN. Adopting the ‘helix models,’ the study looks into specific city plans in Southeast Asia, the ASCN, and experiences with the smart city initiative. 20 The study highlights the disparities in social, economic, and political rights among Southeast Asian states and argues that the presence of states with authoritarian settings generates a more significant challenge for the ASCN. It is then proposed that the smart city conceptualization to be within a ‘system of systems’ framework. 20 In this, “the complex socio-ecological interaction between society and nature and the multi-layered character of governance are acknowledged”. 20

 2.         Paragraph 2: “Consequently, the disparity in economic capacity, social and human rights, and political systems between the Southeast Asian states is perceived as a barrier to achieving the ideal smart city status for ASCN participating cities. With the advancement of technologies proposed as the basis of a smart city, ASEAN is bridging the potential control abuses and heightened cyber surveillance that could be the case in many of the authoritarian nations in the region. As Crumpton’s-led study mentioned, “[…] authoritarian regimes and regimes with authoritarian tendencies seek new and better means for surveilling and controlling their populations”. 20 And that such state systems would impact “[…] participatory and therefore socio-ecologically sustainable urban planning and governance”. 20

**Comment 5:**

 Several design choices compromise the technical robustness of the paper’s primary question—“Are smart cities sustainable?”—and the proposed helix-model framework:

 Construct-to-measurement mismatch (critical). The conclusions of this study on sustainability are primarily derived from country-level indices (HDI, democracy, press freedom, EPI, etc.) and lists of ASCN projects. This approach risks ecological inference problems, where relationships observed at the country level may not apply to city-level patterns or project-level sustainability outcomes. This risk of “ecological fallacy” is well-known in aggregate-level inference work and should be explicitly addressed through design, limitations, and ideally, multi-level data.

 Helix-model integrity problem: Excluding the education helix while retaining the “quintuple helix” language (critical). The article mentions excluding the education stakeholder helix due to an “inconclusive relationship” between ICT projects and top-ranked universities. However, foundational descriptions of the Quintuple Helix model consider the education system as one of the five helices; removing it alters the theoretical framework and necessitates re-specification and justification. If the paper employs a modified helix model, it should be renamed (e.g., “modified quadruple helix” or “adapted helix framework”) and explicitly linked to indicators (construct a diagram and a table that associate each helix with its respective indicator(s), data source, and projected relationship to sustainability). If the education helix is limited, the framework is renamed and the theoretical implications are elucidated, referencing the definitions of the Quintuple Helix).

 The unit of analysis is under-specified. The manuscript shifts between discussing: - ASEAN member states (country-level governance, environmental performance, and development), - ASCN cities (GaWC categorization and project lists), and - “smart city sustainability” as an outcome concept. This could be coherent, but only if the design clearly specifies: (a) the primary unit of analysis, (b) the linking logic between country indices and city/project outcomes, and (c) how alternative explanations are addressed.

 Responses:

 1.         Response 1: I justify in page 7, last paragraph, how the state-level differences and comparisons are applicable in the context of the ASCN cities: “Table 1 below shows how diverse Southeast Asian countries are, showing more significant disparity in the past years. Although the presented data pertain to state actors, there is a strong likelihood that the patterns of democracy, GDP growth, human development, press freedom, ease of doing business, and environmental performance at the state level are also of concern to local and regional governments. This considers that half of Southeast Asian states are non-democratic, suggesting that a hierarchical system will apply to the concerns that government officials focus on.  The indicators consider several dimensions pivotal to the construction of smart cities. First is the 2024 GDP growth, published by the International Monetary Fund, and considers the total value of goods and services produced within the country. 77 Second is the 2022 UN Human Development Index (HDI), which considers education, health, and standard of living. 78 Third is the 2024 Economist Intelligence Unit’s Democracy Index (EIU), which considers civil liberties, the electoral process, political participation & culture, and the functioning of the government. 79 Fourth is the 2024 Reporters Without Borders’ World Press Freedom Index (WPFI), which ranks states based on assessments by non-governmental organizations of journalists' and media freedom. 80 Fifth is the 2024 Yale University’s Environmental Performance Index (EPI), which considers the state of sustainability (climate change performance, environmental health, ecosystem vitality). 81 Last, which differs from the datasets used in the 2021 study helix model assessment on ASCN, is the 2020 World Bank’s EoDB ranks, which rate the difficulty of starting a business, obtaining construction permits, accessing electricity, registering property, and obtaining credit. 82

 2.         Response 2: I introduce several empirical case studies to showcase how the concerns are located in the city level. The following revisions also responds to the comment on the unit of analysis:

 (paragraph 1 page 10): However, what is puzzling is the rising significance of cities in less democratic settings such as Hanoi, Ho Chi Minh City, Phnom Penh, Vientiane, and Myanmar. In the past four years, these four cities have struggled to ensure greater civic rights are maintained. As the ASCN is focused on accelerating the efforts to make these cities ‘smart,’ there seems to be a profound neglect of the political issues in those cities due to this dominant discourse of ‘smart’ development. This raises a substantial concern, as these cities would focus solely on consistency with the ASCN smart city framework and further neglect fundamental urban services. In Vientiane and large cities in Laos, the concern still lies on the issue of restricted civil and political rights, which includes cases of unfair criminal trials, and judicial corruption in the cities (HRW, 2017; OHCHR, 2024). Meanwhile, in Jakarta, those opposing democratic rule have been met with harsh measures to suppress negative sentiments directed at the president (Raditio and Yeremia, 2022; BBC, 2026). These cases go to show that accelerating development that leans to being ‘smart’ does not automatically translate into the provision of basic political and human rights services being delivered. It also shows a strong linkage between state-level political concerns and local-level appreciation towards human rights.

 (paragraph 1, page 11): The ASCN does not consider these capacity disparities. The focus has been on accelerating the ‘smart city’ development of the ASCN’s pilot city projects and on leveraging AI and technology to deliver better public services to the city’s population. However, the complexity is that cities in countries without democratic systems show less appreciation for the environment and lack proper prospects for human development. The updated data from the 2020 study show that the same conclusions have been reached. The disparities will only lead to greater differences among ASCN members, given the absence of sustainability measures in ASCN projects. Perhaps two case studies of concern is in Yangon and Phnom Penh. In Yangon, prosecutions continue amid the military junta’s crackdown on democratic voices (Oponio Juris, 2022; Reuters, 2022). Meanwhile, despite the acceleration of developments in Phnom Penh, this has been met with an increase in money laundering, concerns over foreign debt, and rising unemployment, which shows that smart cities’ focus on transportation services remains unbalanced with more essential services.

 (paragraph 3, page 11): The problem with diverse landscapes such as ASEAN is that everything ‘smart’ can easily deepen authoritarian tendencies and undermine sustainability objectives. 20 Another issue is the lack of acknowledgment that the starting points of these cities are different, with some already building the foundations of smart cities since the 1960s. 90 As a consequence, it is expected that, after observing recent data in the past four years, poor cities would most likely see a more substantial deterioration of sustainability norms, as anticipated in past studies. 92– 94 The case of Hanoi, Vietnam, is interesting, given the numerous criticisms citizens have expressed about the increased number of surveillance cameras in the city. Although intended to protect and maintain order on public roads, there have been concerns that images are being misused to monitor private lives and steal private data (Dan, 2026).

 (paragraph 4, page 11): Combined together, the presented case studies from different ASEAN cities shows an alignment with Crumpton’s concerns over the lack of consideration towards inter-contextual differences. By imposing the ASCN provisions and focusing on targeted smart city elements, this risks the region falling further from sustainability practices that ensure basic services are provided to people.

 3. Response 3: I disagree that a name is required, as the article follows up using more recent empirical case studies and data on Crumpton’s helix model. To emphasize this point, I explain in section 3.2 that the focus of the argument taken from the 2021 study is the idea that disparities in capacities leads to insufficient efforts in promoting sustainability measures in cities. The revised paragraph in parapraph 2 of section 3.2: “The basis of the ASCN's problem is diversity across systems. The smart city approach becomes complex when applied to governments with authoritarian or semi-authoritarian settings, which could lead to further control over populations. 76 For the more democratic nations of Southeast Asia, the problem then becomes to what extent state policymakers consider environmental costs as exceeding development targets. This section argues that old patterns are emerging, with a lack of relationship between citizens and the smart city developments taking place in Southeast Asia. In doing so, the following will explain several indicators that show the diversity between states and cities in Southeast Asia, indicating different starting points among the ASCN members, which risks exacerbating disparities in the delivery of basic urban services. Similar to Crumpton’s 2021 point, the updated empirical data presented between 2020 and 2024 in the two tables below show that concern over non-democratic practices has the potential to negatively affect urban planning and governance, underplay environmental concerns, and neglect improvements in the quality of life of the city’s population. ”

**Comment 6**: Concerns over methods and data availability

 Response 1: I have revised table 1, and no values are labelled as ‘NA.’

 Response 2: Regarding the data, the values and information are publicly available, as stated in the data declaration. Therefore, there is no need to put the data in a separate file and upload the files, as the copyright is retained by the institutions.
